# Exploring the Relationship Between Psychiatric Traits and the Risk of Mouth Ulcers Using Bi-Directional Mendelian Randomization

**DOI:** 10.3389/fgene.2020.608630

**Published:** 2020-12-16

**Authors:** Kai Wang, Lin Ding, Can Yang, Xingjie Hao, Chaolong Wang

**Affiliations:** ^1^Key Laboratory for Environment and Health, Department of Epidemiology and Biostatistics, School of Public Health, Tongji Medical College, Huazhong University of Science and Technology, Wuhan, China; ^2^Department of Mathematics, The Hong Kong University of Science and Technology, Hong Kong, China

**Keywords:** psychiatric traits, mouth ulcers, Mendelian randomization, causality, GWAS summary statistics

## Abstract

**Background:**

Although the association between mouth ulcers and psychiatric traits has been reported by observational studies, their causal relationship remains unclear. Mendelian randomization (MR), powered by large-scale genome-wide association studies (GWAS), provides an opportunity to clarify the causality between mouth ulcers and psychiatric traits.

**Methods:**

We collected summary statistics of mouth ulcers (sample size *n* = 461,106) and 10 psychiatric traits from the largest publicly available GWAS on Europeans, including anxiety disorder (*n* = 83,566), attention deficit/hyperactivity disorder (*n* = 53,293), autism spectrum disorder (*n* = 46,350), bipolar disorder (*n* = 51,710), insomnia (*n* = 1,331,010), major depressive disorder (*n* = 480,359), mood instability (*n* = 363,705), neuroticism (*n* = 168,105), schizophrenia (*n* = 105,318), and subjective wellbeing (*n* = 388,538). We applied three two-sample bi-directional MR analysis methods, namely the Inverse Variance Weighted (IVW) method, the MR pleiotropy residual sum and outlier (MR-PRESSO) method, and the weighted median method, to assess the causal relationship between each psychiatric trait and mouth ulcers.

**Results:**

We found significant effects of autism spectrum disorder, insomnia, major depressive disorder, and subjective wellbeing on mouth ulcers, with the corresponding odds ratio (*OR*) from the IVW method being 1.160 [95% confidence interval (*CI*): 1.066–1.261, *P* = 5.39 × 10^–4^], 1.092 (1.062–1.122, *P* = 3.37 × 10^–10^), 1.234 (1.134–1.342, *P* = 1.03 × 10^–6^), and 0.703 (0.571–0.865, *P* = 8.97 × 10^–4^), respectively. We also observed suggestive evidence for mood instability to cause mouth ulcers [IVW, OR = 1.662 (1.059–2.609), *P* = 0.027]. These results were robust to weak instrument bias and heterogeneity. We found no evidence on causal effects between other psychiatric traits and mouth ulcers, in either direction.

**Conclusion:**

Our findings suggest a protective effect of subjective wellbeing and risk effects of autism spectrum disorder, insomnia, major depressive disorder, and mood instability on mouth ulcers. These results clarify the causal relationship between psychiatric traits and the development of mouth ulcers.

## Introduction

A mouth ulcer (also termed oral ulceration) is an ulcer that occurs on the mucous membrane of the oral cavity, involving damage to both epithelium and lamina propria (Scully, 2008; Tugrul et al., 2016). Mouth ulcers are prevalent worldwide, affecting nearly 25% of young adults and a higher proportion of children (Scully, 2006; Paleri et al., 2010; Tugrul et al., 2016; Dudding et al., 2019). Although mouth ulcers do not pose a substantial health burden, they can interfere with daily activities (such as speaking or swallowing) and have detrimental effects on individual quality of life, overall wellbeing, and social interaction (Huling et al., 2012; Almoznino et al., 2014; Al-Omiri et al., 2015). Furthermore, mouth ulcers are one of the common clinical signals of several serious diseases, such as oral cancer, gastrointestinal diseases, and human immunodeficiency virus infection (Paleri et al., 2010; Bilodeau and Lalla, 2019). Besides, mouth ulcers have been reported to associate with head and neck cancer, pancreatic cancer, breast cancer, and prostate cancer by a recent epidemiology study (Qin et al., 2018).

The high prevalence of mouth ulcers and its undesired impact on life quality have motivated numerous studies on the etiology and efficient therapy of this disease. Recurrent aphthous stomatitis (RAS) is the most common cause, followed by local trauma, malignancy, and infection (Paleri et al., 2010; Gavic et al., 2014; Al-Omiri et al., 2015; Bilodeau and Lalla, 2019). Nevertheless, the pathogenesis of mouth ulcers is still poorly understood. Psychiatric disorders are potential risk factors for mouth ulcers, as suggested by observational studies. For example, patients with depression and anxiety are more likely to develop mouth ulcers according to a series of observational studies (Huling et al., 2012; Alshahrani and Baccaglini, 2014; Ma et al., 2015; Ge, 2018); high levels of psychological stress were found in mouth ulcers patients (Gallo Cde et al., 2009); depression and neuroticism were genetically correlated with mouth ulcers (Dudding et al., 2019); and a transitory rise in salivary cortisol and/or changes in immunoregulatory activity caused by psychiatric disorders were linked to mouth ulcers (MacGregor et al., 1969; Redwine et al., 2003; Slebioda and Dorocka-Bobkowska, 2019). These observations together lead to a hypothesis that psychiatric disorders may trigger mouth ulcers. Nevertheless, the causal relationship between psychiatric traits and mouth ulcers remains largely unclear.

With the development of large-scale GWAS and Mendelian randomization (MR), causal inference between complex traits and diseases has become possible (Lawlor et al., 2008; Hartwig et al., 2017). The MR approach uses genetic variants, such as single nucleotide polymorphisms (SNPs), associated with a modifiable exposure (e.g., a psychiatric trait) as the instrumental variables (IVs) to estimate the causality between this exposure and an outcome of interest (e.g., mouth ulcers) (Lawlor et al., 2008). The basic idea is that SNPs associated with the exposure, which were randomly passed from parents to offsprings during meiosis irrespective of confounders, would also be associated with the outcome if the exposure is causally associated with the outcome. To ensure the validity of MR for causal inference, the IVs need to satisfy three model assumptions: (a) associated with the exposure (the relevance assumption); (b) independent of any confounder of the exposure-outcome association (the independence assumption); and (c) only affect the outcome through the exposure (the exclusion restriction assumption) (Lawlor et al., 2008; Hartwig et al., 2016). Recent studies have found that the exclusion restriction assumption may be too strong given the polygenic architecture of complex traits/disease and the ubiquity of pleiotropy (Zhao et al., 2019). Instead, an alternative weaker assumption named Instrument Strength Independent of Direct Effect (InSIDE) has been proposed (Bowden et al., 2015). This assumption allows for the direct effects of IVs on the outcome, assuming that genetic associations with the exposure are independent of the direct effects (Bowden et al., 2015). Two-sample MR refers to the application of MR on GWAS summary statistics of the exposure and the outcome from two independent samples, which can overcome the winner’s curse and maximize the statistical power (Burgess et al., 2016). Further information about the assumptions and interpretations of MR can be found elsewhere (Haycock et al., 2016; Zheng et al., 2017; Davies et al., 2018).

As the pathogenesis of mouth ulcers is complicated, identification of causal risk factors will be useful to facilitate both the prevention and treatment of the disease. In this study, we aim to systematically investigate the causal relationship between mouth ulcers and psychiatric traits. We conducted two-sample bi-directional MR analyses using publicly available GWAS summary statistics of mouth ulcers and 10 psychiatric traits, including anxiety disorder, attention deficit/hyperactivity disorder (ADHD), autism spectrum disorder (ASD), bipolar disorder (BIP), insomnia, major depressive disorder (MDD), mood instability, schizophrenia (SCZ), neuroticism, and subjective wellbeing (Pardiñas et al., 2018; Turley et al., 2018; Wray et al., 2018; Demontis et al., 2019; Grove et al., 2019; Jansen et al., 2019; Purves et al., 2019; Stahl et al., 2019; Ward et al., 2020).

## Materials and Methods

### Data Collection

We collected GWAS summary statistics of eight psychiatric traits and mouth ulcers from published studies with the largest sample sizes of European ancestry (Table 1). In addition, we only obtained summary statistics of significant associated SNPs for anxiety disorders and mood instability due to restricted access to the GWAS summary statistics of these two traits. GWAS on mouth ulcers were based on the UK Biobank (UKB), in which all participants were asked about their oral health in the baseline questionnaire. “Mouth ulcers (yes/no)” was defined as having mouth ulcers within the last year. Supplementary Table S1 lays out the definitions of 10 psychiatric traits.

**TABLE 1:** Description of GWAS consortiums used for each trait.

Trait	Population	Sample size (cases/controls)	Sample overlap^$^	Data source	References
Anxiety disorders	Europeans	25,453/58,113	18.2%	UKB	Purves et al., 2019
ADHD	96% Europeans	19,099/34,194	0	PGC	Demontis et al., 2019
ASD	Europeans	18,381/27,969	0	PGC	Grove et al., 2019
BIP	Europeans	20,352/31,358	0	PGC	Stahl et al., 2019
Insomnia	Europeans	397,972/933,038	29.0%	UKB, 23andMe	Jansen et al., 2019
MDD	Europeans	135,458/344,901	6.5%	UKB, 23andMe, PGC29, deCODE, GenScot, GERA, iPSYCH	Wray et al., 2018
Mood instability	Europeans	157,039/206,666	78.9%	UKB	Ward et al., 2020
Neuroticism	Europeans	168,105	22.5%	UKB, GPC	Turley et al., 2018
SCZ	Europeans	40,675/64,643	0	CLOZUK, PGC	Pardiñas et al., 2018
Subjective wellbeing	Europeans	388,538	8.8%	UKB, 23andMe, SSGAC	Turley et al., 2018
Mouth ulcers	Europeans	47,079/414,027	–	UKB	Dudding et al., 2019

**ADHD, attention deficit/hyperactivity disorder; ASD, autism spectrum disorder; BIP, bipolar disorder; MDD, major depressive disorder; SCZ, Schizophrenia; UKB, the UK Biobank; 23andMe, 23andMe company; PGC29, the Psychiatric Genomics Consortium, 29 European samples; deCODE, deCODE Genetics company; GenScot, Generation Scotland: Scottish Family Health Study; GERA, Genetic Epidemiology Research on Adult Health and Aging Study; iPSYCH, The Lundbeck Foundation Initiative for Integrative Psychiatric Research; SSGAC, Social Science Genetics Association Consortium; PGC, the Psychiatric Genomics Consortium; GPC, the Genetics of Personality Consortium. ^$^The overlapping sample size divided by the larger sample size of the corresponding psychiatric trait and mouth ulcers. For the two quantitative traits, neuroticism and subjective wellbeing, the total sample size was present.**

### Patient and Public Involvement

Because this study used published GWAS summary statistics available in the public domain, specific ethical review or consent from study participants was not sought.

### Statistical Analyses

**FIGURE 1:**
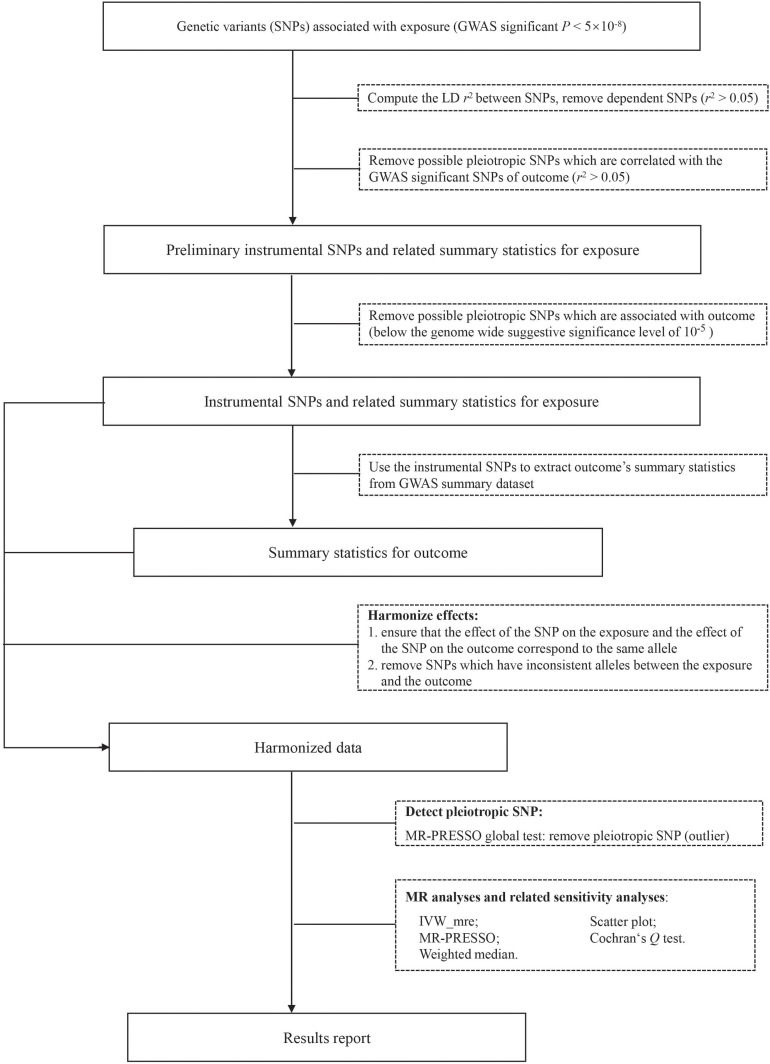
The analysis flowchart of this study. IVW_mre, inverse variance weighted multiplicative random effects; MR-PRESSO, Mendelian randomization pleiotropy residual sum and outlier.

The overall workflow of our analyses was summarized in Figure 1. We took the following steps to choose valid instrumental SNPs given the assumptions of MR. Firstly, candidate IVs were restricted to those of genome-wide significant association (*P* < 5 × 10^–8^) with the exposure (e.g., a psychiatric trait). Secondly, we pruned the candidate IVs to independent SNPs (*r*^2^ < 0.05, window size = 1 Mb), keeping those with the smallest *P*-values, based on linkage disequilibrium (LD) calculated from the 1000 Genomes Project Phase 3 European dataset using PLINK v.1.90 (Chang et al., 2015; Consortium, 2015). Thirdly, because bi-directional MR assumes no overlap or LD between the IVs for the exposure and the outcome (Davey Smith and Hemani, 2014), we excluded SNPs in LD (*r*^2^ > 0.05) with the significant SNPs for mouth ulcers. Finally, we removed potential pleiotropic SNPs by excluding SNPs of suggestive association (*P* < 10^–5^) with mouth ulcers (Au Yeung et al., 2017; Zeng and Zhou, 2019). The remaining SNPs were used as valid IVs to conduct MR analyses. Valid IVs for all exposure-outcome pairs are listed in Supplementary Tables S2–S19. For each IV, we computed the *F* statistic to quantify whether it was strongly associated with the exposure (Lawlor et al., 2008). For multiple IVs, we computed the *F* statistic as the mean of the *F* statistics of individual IVs and the 95% confidence interval (*CI*) by 10,000 bootstraps (Burgess et al., 2016).

Given an IV, the causal effect of exposure (*X*) on outcome (*Y*), β_*XY*_ can be estimated as ${\hat{\beta }}_{I\,V}={\hat{\beta }}_{Z\,Y}/{\hat{\beta }}_{Z\,X}$ where ${\hat{\beta }}_{Z\,Y}$ represents the effect of the IV (*Z*) on the outcome (*Y*), ${\hat{\beta }}_{Z\,X}$ represents the effect of the IV (*Z*) on the exposure (*X*), and the variance of ${\hat{\beta }}_{I\,V}$ can be estimated by the delta method (Thomas et al., 2007). In the presence of multiple IVs (e.g., multiple instrumental SNPs), several methods have been proposed to estimate β_*XY*_ under different assumptions. In this study, we used three different methods, namely the Inverse Variance Weighted (IVW) method (Burgess et al., 2013), the MR pleiotropy residual sum and outlier (MR-PRESSO) method (Verbanck et al., 2018), and the weighted median method (Bowden et al., 2016a).

Briefly, the IVW estimate is the IVW average of ${\hat{\beta }}_{I\,V}$, assuming all SNPs are valid IVs or the overall bias is zero (balanced pleiotropy) (Bowden et al., 2016b). We performed multiplicative random effects IVW to account for potential heterogeneity, which is measured by the Cochran’s *Q* statistic (Hemani et al., 2018a). The IVW method is equivalent to fitting a weighted linear regression with no intercept of ${\hat{\beta }}_{Z\,Y}$ on ${\hat{\beta }}_{Z\,X}$ where the weight is the inverse variance of ${\hat{\beta }}_{Z\,Y}$ and the estimated regression slope is the estimated causal effect of the exposure on the outcome (β_*XY*_).

The MR-PRESSO method is designed to correct for horizontal pleiotropy, in which the IV acts on the outcome via a pathway other than through the exposure. MR-PRESSO is based on the IVW regression framework and detects IVs of horizontal pleiotropy as outliers in the regression. In particular, MR-PRESSO implements a global test based on the leave-one-out approach to test for the existence of horizontal pleiotropy and an outlier test to detect specific SNPs with horizontal pleiotropy. MR-PRESSO provides the final IVW estimate after removing outlier IVs (Verbanck et al., 2018).

Finally, the weighted median method uses the inverse variance of ${\hat{\beta }}_{I\,V}$ as weight to construct the empirical distribution of ${\hat{\beta }}_{I\,V}$, and derives the final estimate by taking the median (Bowden et al., 2016a). The confidence interval of the weighted median estimate is obtained by a parametric bootstrap method. This method can provide a consistent estimate as long as at least 50% of the weight comes from the valid IVs.

We displayed the scatter plot of genetic effect on the outcome (${\hat{\beta }}_{Z\,Y}$) vs. genetic effect on the exposure (${\hat{\beta }}_{Z\,X}$) for each IV to facilitate the identification of possible heterogeneity and the illustration of causal effects. We used mRnd^1^ to calculate *post hoc* statistical power. With a Bonferroni-corrected significance level of 2.8 × 10^–3^ (α = 0.05/18, correcting 18 exposure-outcome paired tests), we estimated the required OR of exposure on outcome (in the unit of per standard deviation increment in exposure) to achieve 80% statistical power given the summary statistics (Brion et al., 2013). A causal effect of an exposure on the outcome is concluded if the effect estimates agree in direction and magnitude among MR methods, pass the Bonferroni-corrected significance threshold of 2.8 × 10^–3^ in the IVW method, and show no evidence of heterogeneity in the Cochran’s *Q*-test and MR-PRESSO global test. Findings with *P*-values between 0.05 and 2.8 × 10^–3^ were deemed suggestive evidence of causality. Analyses were performed with TwoSampleMR and MR-PRESSO packages in R version 3.5.3 (Hemani et al., 2018b; Verbanck et al., 2018; Team RC, 2019).

## Results

### Psychiatric Traits Predicting Mouth Ulcers

**FIGURE 2:**
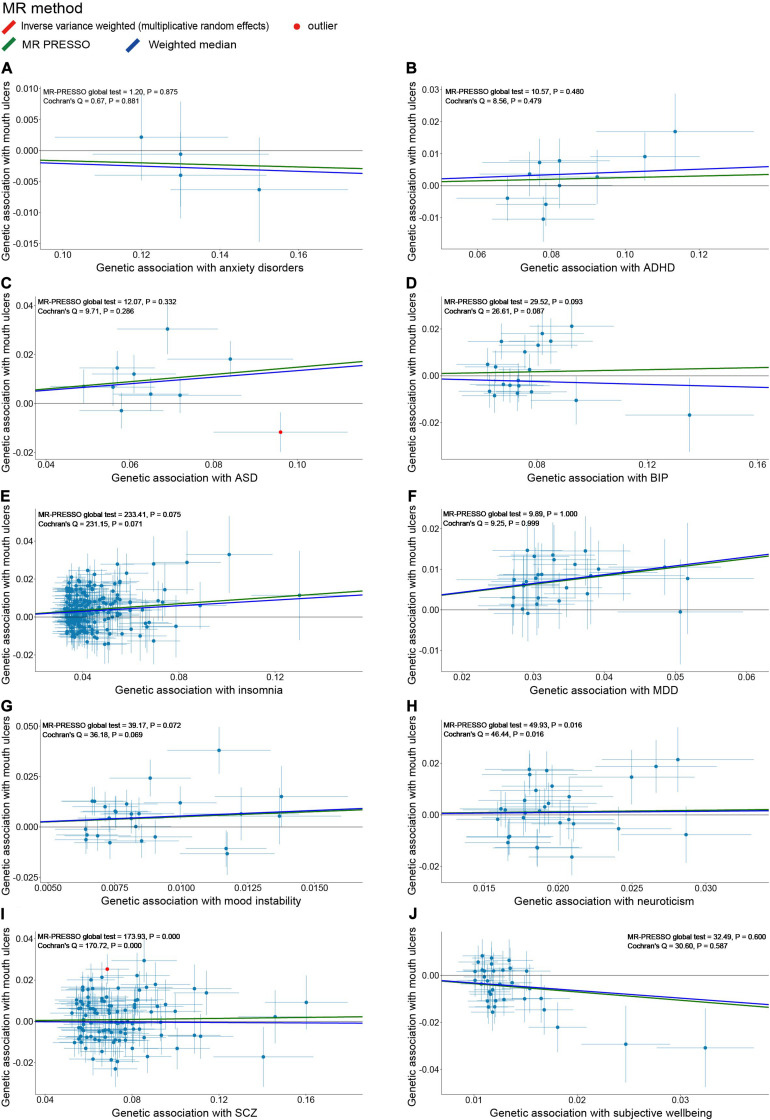
Scatter plots of genetic associations with mouth ulcers (outcome) vs. genetic associations with 10 psychiatric traits (exposure) for all the valid IVs. **(A)** anxiety disorders; **(B)** ADHD; **(C)** ASD; **(D)** BIP; **(E)** insomnia; **(F)** MDD; **(G)** mood instability; **(H)** neuroticism; **(I)** SCZ; **(J)** subjective wellbeing. Each dot corresponds to one genetic variant, with corresponding standard error bars of its association with psychiatric trait (horizontal) and mouth ulcers (vertical); solid red dot represents the pleiotropic SNP (outlier) identified by MR-PRESSO global test; the solid lines illustrate estimations of the causal effect after excluding outlier SNP, colored by different colors with different MR methods. The horizontal gray solid line indicates no effect. In this study, the causal effect estimations from the IVW_mre and MR-PRESSO are consistent, such that the red line (IVW_mre) is covered by the green line (MR-PRESSO), and no red line could be observed.

**FIGURE 3:**
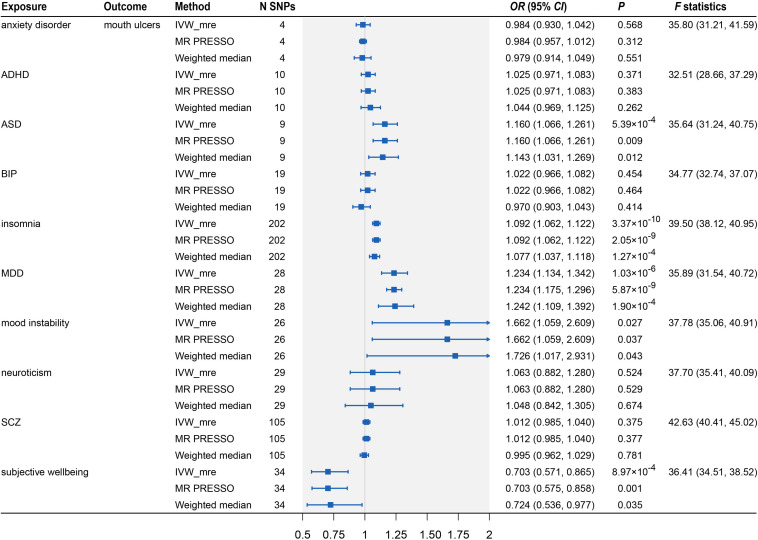
Two-sample Mendelian randomization analyses showing the effect estimates of 10 psychiatric traits on mouth ulcers. ADHD, attention deficit/hyperactivity disorder; ASD, autism spectrum disorder; BIP, bipolar disorder; MDD, major depressive disorder; SCZ, schizophrenia; IVW_mre, inverse variance weighted with multiplicative random effects; MR-PRESSO, Mendelian randomization pleiotropy residual sum and outlier; N SNP, number of the instrumental SNPs used to conduct MR analyses; Effect estimates express the change in odds ratio (*OR*) per standard deviation (SD) increment in psychiatric traits, error bars indicate 95% confidence intervals.

After the IV selection process, we displayed the genetic associations with mouth ulcers over genetic associations with psychiatric traits for the valid IVs (Figure 2). By the MR-PRESSO outlier test, we detected two outlier SNPs (solid red dots in Figures 2C,I): one each for ASD and SCZ. After removing these two SNPs, all three MR methods agreed well in fitting the linear relationship between the genetic effect sizes on mouth ulcers and each of the psychiatric traits (colored solid lines in Figure 2). Estimates of the causal effects of 10 psychiatric traits on mouth ulcers were presented in Figure 3. We found that ASD, insomnia, and MDD have significant risk effects and subjective wellbeing has significant protective effect on mouth ulcers. The corresponding effect sizes from the IVW method were OR = 1.160 (95% CI: 1.066–1.261, *P* = 5.39 × 10^–4^), 1.092 (1.062–1.122, *P* = 3.37 × 10^–10^), 1.234 (1.134–1.342, *P* = 1.03 × 10^–6^), and 0.703 (0.571–0.865, *P* = 8.97 × 10^–4^) for ASD, insomnia, MDD, and subjective wellbeing, respectively. There was suggestive evidence for risk effect of mood instability on mouth ulcers (IVW, OR = 1.662, 95% CI: 1.059–2.609, *P* = 0.027). All the *F* statistics were greater than 32, indicating robust causal estimates against the weak instrument bias (Figure 3). We confirmed that these estimated effect sizes were close to or above the threshold to achieve 80% statistical power given the available summary statistics (Supplementary Table S20). Importantly, the MR-PRESSO global test and Cochran *Q*-test suggested no heterogeneity or pleiotropic effect (Supplementary Table S22). We found no evidence of causal effects on mouth ulcers from all three MR methods for the remaining five psychiatric traits (anxiety disorder, ADHD, BIP, neuroticism, and SCZ, Figures 2, 3 and Supplementary Tables S20, S22).

### Mouth Ulcers Predicting Psychiatric Traits

**FIGURE 4:**
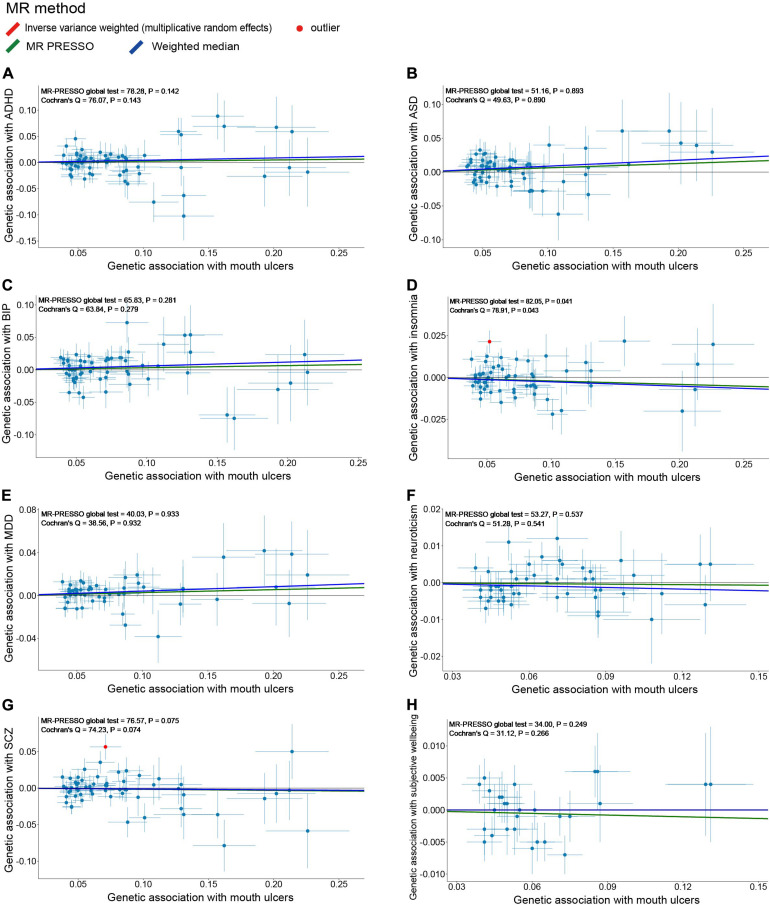
Scatter plots of genetic associations with eight psychiatric traits (outcome) vs. genetic associations with mouth ulcers (exposure) for all the valid IVs. **(A)** ADHD; **(B)** ASD; **(C)** BIP; **(D)** insomnia; **(E)** MDD; **(F)** neuroticism; **(G)** SCZ; **(H)** subjective wellbeing. Each dot corresponds to one genetic variant, with corresponding standard error bars of its association with mouth ulcers (horizontal) and psychiatric trait (vertical); solid red dot represents the pleiotropic SNP (outlier) identified by MR-PRESSO global test; the solid lines illustrate estimations of the causal effect after excluding outlier SNP, colored by different colors with different MR methods. The horizontal gray solid line indicates no effect. In this study, the causal effect estimations from the IVW_mre and MR-PRESSO are consistent, such that the red line (IVW_mre) is covered by the green line (MR-PRESSO), and no red line could be observed.

**FIGURE 5:**
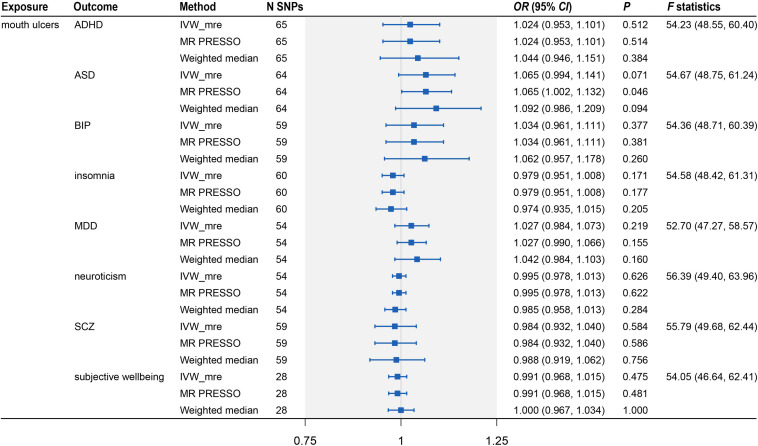
Two-sample Mendelian randomization analysis showing the effect of mouth ulcers on eight psychiatric traits. ADHD, attention deficit/hyperactivity disorder; ASD, autism spectrum disorder; BIP, bipolar disorder; MDD, major depressive disorder; SCZ, schizophrenia; IVW_mre, inverse variance weighted with multiplicative random effects; MR-PRESSO, Mendelian randomization pleiotropy residual sum and outlier; N SNP, number of the instrumental SNPs used to conduct MR analyses; Effect estimates express the change in odds ratio (*OR*) per standard deviation (*SD*) increment in mouth ulcers, error bars indicate 95% confidence intervals.

Because the summary statistics of anxiety disorders and mood instability were only available for significantly associated SNPs, we could not perform MR analyses of mouth ulcers on these two traits. For the remaining eight psychiatric traits, we displayed their genetic effect sizes vs. the genetic effect sizes on mouth ulcers for the valid IVs (Figure 4). Two outlier SNPs (solid red dots in Figures 4D,G) were detected by the MR-PRESSO outlier test and excluded subsequently. Although the instrumental SNPs could explain a substantial amount of phenotypic variance (≥ 0.9% for all eight traits, Supplementary Table S21) and the *F* statistics indicated strong instrumental effects (all *F* > 52), we found no significant evidence of causal effects of mouth ulcers on these psychiatric traits (Figures 4, 5). The only suggestive evidence was given by the MR-PRESSO method for mouth ulcers on ASD (*OR* = 1.065, 95% CI: 1.002–1.132, *P* = 0.046). The effect estimates for mouth ulcers on ASD were 1.065 (0.994–1.141, *P* = 0.071) and 1.092 (0.986–1.209, *P* = 0.094) by the IVW method and the weighted median method, respectively (Figure 5). Furthermore, these effect estimates were below the threshold to achieve 80% statistical power, suggesting a high potential for false discoveries (Supplementary Table S21). No heterogeneity or directional pleiotropy was indicated by the MR-PRESSO global test and Cochran *Q*-test (Supplementary Table S23).

## Discussion

Psychiatric disorders have been suggested to associate with mouth ulcers by observational studies. We performed two-sample bi-directional MR analyses to explore the causality between 10 psychiatric traits (anxiety disorder, ADHD, ASD, BIP, insomnia, MDD, mood instability, neuroticism, SCZ, and subjective wellbeing) and mouth ulcers based on summary statistics of the largest available GWAS to date. Our analyses suggested that ASD, insomnia, MDD, and mood instability have risk effects and subjective wellbeing has a protective effect on mouth ulcers, whereas mouth ulcers have no significant effect on any of these psychiatric traits. Our analyses were well-powered and did not suffer from weak instrumental bias according to the *F* statistics. The MR-PRESSO global test, Cochran’s *Q*-test, and scatter plots indicated no directional pleiotropy or heterogeneity.

It has been pointed out that stress, depression, and anxiety are associated with mouth ulcers by a cross-sectional study (Alshahrani and Baccaglini, 2014). A recent study, based on linkage disequilibrium score regression analysis, also found a significant genetic correlation (correlation coefficient = 0.24, *P* = 5.73 × 10^–7^) between depression and mouth ulcers in Europeans (Dudding et al., 2019). Using bi-directional MR analyses, we tested these observational results and confirmed that MDD has a causal effect on mouth ulcers. However, inconsistent findings were observed for anxiety; our analyses did not support a causal relationship between anxiety and mouth ulcers. The relatively small sample size (*n* = 83,566) and only 0.2% of phenotype variation explained by the four IVs of anxiety may explain the null finding. While the precise mechanism linking depression to mouth ulcers is not well understood, the immune system or inflammatory response is suggested to be involved (Al-Omiri et al., 2012; Huling et al., 2012; Alshahrani and Baccaglini, 2014). Depression can increase the number of leukocytes, which exhibit increased motility and enhanced adhesion to endothelial cells and thus induce endothelial dysfunction and mouth ulcers ultimately (Gavic et al., 2014; Demir et al., 2015; Qin et al., 2018). Besides, a serotonin transporter gene polymorphism (*5-HTTLPR*), which is commonly found in depressed patients, is also significantly enriched in patients with mouth ulcers (Victoria et al., 2005). Further functional experiments are required to clarify the mechanistic link between MDD and mouth ulcers.

Many observational studies have reported positive associations between stress and mouth ulcers (Al-Omiri et al., 2012; Huling et al., 2012; Ma et al., 2015; Ge, 2018). For example, ulceration is exacerbated during examination periods and lessened during periods of vacation for students (Scully, 2013). Meanwhile, stress is well known to correlate with mood instability and subjective wellbeing (Schneiderman et al., 2005; Atanes et al., 2015; Berrios et al., 2016; Gillett and Crisp, 2017; Faurholt-Jepsen et al., 2019). Our study suggested that mood instability and subjective wellbeing are causally associated with mouth ulcers using several MR methods. Stress is thought to affect multiple immune system components including the distribution and proliferation of lymphocytes and natural killer cells and production of cytokines and antibodies (Huling et al., 2012). Stressful situations can cause a transitory increase of salivary cortisol and stimulate immunoregulatory activity by increasing the number of leukocytes in inflammatory sites, which are often observed during the pathogenesis of mouth ulcers (Albanidou-Farmaki et al., 2008; Gallo Cde et al., 2009; Al-Omiri et al., 2015). However, the exact mechanism about how stress-related mood instability and subjective wellbeing trigger mouth ulcers remains to be elucidated.

Consistent with previous observational studies (Ma et al., 2015; Du et al., 2018), we also found that insomnia has a causal effect to increase the risk of mouth ulcers. Insomnia will lead to late bedtime, which can disturb the secretion of hormones, such as growth hormone, cortisol, and adrenocorticotropic hormone (Ma et al., 2015). The reduced secretion of growth hormone can promote the occurrence of mouth ulcers and delay healing (Brandenberger, 2004; Dioufa et al., 2010; Lee et al., 2010; Smaniotto et al., 2011). Insufficient secretion of cortisol and adrenocorticotropic hormone may also increase inflammation and allergic reactions and facilitate the occurrence of mouth ulcers (MacGregor et al., 1969; Bierwolf et al., 2000; Sakamoto et al., 2013; Gavic et al., 2014). Hormonal factors are capable of altering the thickness of the mucosa, which is an important factor in mouth ulcers (Neville et al., 2008; Scully, 2013).

It is worth noting that MR uses genetic variants as the IVs such that its causal effect estimate represents the average effect of lifetime exposure on the outcome (Holmes et al., 2017). Most of the psychiatric traits we studied were clinically diagnosed long-term disorders (Supplementary Table S1), but their clinical symptoms might be time-dependent. For example, patients with anxiety disorders might present different levels of anxiety across time periods. Hence, the risk of developing mouth ulcers is also likely to be time-dependent if the anxiety symptom is causal. More caution needs to be taken when interpreting causal effect sizes derived from MR analysis in clinical practice.

Our bi-directional MR analyses had important strengths. Firstly, using randomly allocated genetic variants as IVs, we could reduce the potential impacts of conventional confounders and reverse causality, which are common in observational studies. Secondly, the SNP-exposure and SNP-outcome estimates we used were derived from studies of the largest sample sizes to date (ranging from 46,350 to 1,331,010 individuals), allowing credible causal inference between psychiatric traits and mouth ulcers in the European population. Thirdly, by utilizing a bi-directional MR design, we evaluated the causal relationship between two traits simultaneously and could assess the causal direction more confidently. Finally, our conclusions were drawn based on comprehensive analyses involving 10 psychiatric traits, three credible MR methods, and several heterogeneity tests to prevent possible pleiotropic bias.

There were also some limitations in our study. First, our analysis did not distinguish different types of mouth ulcers, because mouth ulcers in UKB were inferred from the questionnaire rather than clinical examination. Given that most of the significant variants from UKB have been validated in independent samples, including three specific to RAS, the major type of mouth ulcers (Bilodeau and Lalla, 2019), while other types of ulcers, such as traumatic mouth ulcers, are less likely to be genetic (Dudding et al., 2019), our findings are expected to largely reflect the causality between psychiatric traits and RAS. Second, the sample overlapping between GWAS of mood instability and mouth ulcers was as large as 78.9%, which violated the assumption of two-sample MR. Nevertheless, the *F* statistic was large enough (*F* = 37.78, 95% CI: 35.06–40.91), suggesting that the sample overlapping would not materially affect the causal inference (Burgess et al., 2016). Third, consistent with findings in other MR studies involving of psychiatric traits, the effect sizes of genetic variants on psychiatric traits were estimated with large standard errors (Figure 2), indicating difficulty to accurately measure these traits (Wootton et al., 2018; Vermeulen et al., 2019). For this reason, we did not use the MR-Egger method, because it assumes that the associations between IVs and the exposure are precisely estimated or have a wide spread (Bowden et al., 2016b; Burgess and Thompson, 2017). Fourth, cautions are needed when generalizing our findings, which were derived from data of European population, to non-European populations, because different environmental factors might have substantial impacts on psychiatric traits and mouth ulcers. Lastly, we did not consider sex-specific effects, which might differ for psychiatric traits and mouth ulcers due to differences in hormone levels. Because GWAS summary statistics we collected were not stratified by sex, we could not perform sex-specific analyses to validate different sex-specific causal effects of psychiatric traits on mouth ulcers observed in epidemiological studies (Huling et al., 2012; Slebioda and Dorocka-Bobkowska, 2019).

## Conclusion

In conclusion, utilizing large-scale GWAS summary statistics and two-sample bi-directional MR analyses, our study provides causal evidence on the risk role of ASD, insomnia, MDD, and mood instability, and the protective role of subjective wellbeing on mouth ulcers in the European population. Future work is needed to understand the biological pathways from psychiatric traits to mouth ulcers.

## Data Availability Statement

The original contributions presented in the study are included in the article/Supplementary Material, further inquiries can be directed to the corresponding author/s.

## Author Contributions

XH and CW conceived and supervised the study. KW and LD collected and analyzed the data. KW, XH, and CW wrote the manuscript with inputs from CY. All authors have reviewed and approved the final manuscript.

## Conflict of Interest

The authors declare that the research was conducted in the absence of any commercial or financial relationships that could be construed as a potential conflict of interest.

## Footnotes

1 http://cnsgenomics.com/shiny/mRnd/

## Abbreviations

GWAS: genome-wide association studies
LD: linkage disequilibrium
SNP: single nucleotide polymorphisms
MR: Mendelian randomization
IV: instrumental variable
InSIDE: Instrument Strength Independent of Direct Effect
IVW: Inverse Variance Weighted
MR-PRESSO: MR pleiotropy residual sum and outlier
OR: odds ratio
CI: confidence interval
SD: standard deviation
UKB: UK Biobank
23andMe: 23andMe company
PGC29, the Psychiatric Genomics Consortium: 29 European samples
deCODE: deCODE Genetics company
GenScot: Generation Scotland: Scottish Family Health Study
GERA: Genetic Epidemiology Research on Adult Health and Aging Study
iPSYCH: The Lundbeck Foundation Initiative for Integrative Psychiatric Research
SSGAC: Social Science Genetics Association Consortium
PGC: the Psychiatric Genomics Consortium
GPC: the Genetics of Personality Consortium
RAS: Recurrent aphthous stomatitis
ADHD: attention deficit/hyperactivity disorder
ASD: autism spectrum disorder
BIP: bipolar disorder
MDD: major depressive disorder
SCZ: schizophrenia.
