# Supplementary material for: Exploring the Relationship Between Psychiatric Traits and the Risk of Mouth Ulcers Using Bi-Directional Mendelian Randomization
Source: Front Genet. 2020 Dec 16;11:608630. doi: 10.3389/fgene.2020.608630 (PMC7793678; doi:10.3389/fgene.2020.608630)
Supplement: Supplementary file 1 [file Data_Sheet_1.docx]

**Exploring the relationship between psychiatric traits and the risk of mouth ulcers using bi-directional Mendelian randomization**

Kai Wang^1^, Lin Ding^1^, Can Yang^2^, Xingjie Hao^1,§^, Chaolong Wang^1,§^

^1^ Department of Epidemiology and Biostatistics, Key Laboratory for Environment and Health, School of Public Health, Tongji Medical College, Huazhong University of Science and Technology, Wuhan, China.

^2^ Department of Mathematics, The Hong Kong University of Science and Technology, Hong Kong SAR, China.

^§^ Correspondence: [chaolong@hust.edu.cn](mailto:chaolong@hust.edu.cn), +86-133-9606-9645 (C.W.); [xingjie@hust.edu.cn](mailto:xingjie@hust.edu.cn), +86-158-0713-3891 (X.H.)

| **Index of Supplementary Tables** |  |
| --- | --- |
| [**Table S1.** Description of GWAS phenotype for each trait.](#S1) | Page 3 |
| [**Table S2.** 4 valid instrumental variables used for Mendelian randomization analysis of anxiety disorder (Exposure) on mouth ulcers (Outcome).](#S2) | Page 4 |
| [**Table S3.** 10 valid instrumental variables used for Mendelian randomization analysis of attention deficit/hyperactivity disorder (Exposure) on mouth ulcers (Outcome).](#S3) | Page 5 |
| [**Table S4.** 10 valid instrumental variables used for Mendelian randomization analysis of autism spectrum disorder (Exposure) on mouth ulcers (Outcome).](#S4) | Page 6 |
| [**Table S5.** 19 valid instrumental variables used for Mendelian randomization analysis of bipolar disorder (Exposure) on mouth ulcers (Outcome).](#S5) | Page 7 |
| [**Table S6.** 202 valid instrumental variables used for Mendelian randomization analysis of insomnia (Exposure) on mouth ulcers (Outcome).](#S6) | Page 8 |
| [**Table S7.** 28 valid instrumental variables used for Mendelian randomization analysis of major depressive disorder (Exposure) on mouth ulcers (Outcome).](#S7) | Page 13 |
| [**Table S8.** 26 valid instrumental variables used for Mendelian randomization analysis of mood instability (Exposure) on mouth ulcers (Outcome).](#S8) | Page 14 |
| [**Table S9.** 29 valid instrumental variables used for Mendelian randomization analysis of neuroticism (Exposure) on mouth ulcers (Outcome).](#S9) | Page 15 |
| [**Table S10.** 106 valid instrumental variables used for Mendelian randomization analysis of schizophrenia (Exposure) on mouth ulcers (Outcome).](#S10) | Page 16 |
| [**Table S11.** 34 valid instrumental variables used for Mendelian randomization analysis of subjective wellbeing (Exposure) on mouth ulcers (Outcome).](#S11) | Page 19 |
| [**Table S12.** 65 valid instrumental variables used for Mendelian randomization analysis of mouth ulcers (Exposure) on attention deficit/hyperactivity disorder (Outcome).](#S12) | Page 20 |
| [**Table S13.** 64 valid instrumental variables used for Mendelian randomization analysis of mouth ulcers (Exposure) on autism spectrum disorder (Outcome).](#S13) | Page 22 |
| [**Table S14.** 59 valid instrumental variables used for Mendelian randomization analysis of mouth ulcers (Exposure) on bipolar disorder (Outcome).](#S14) | Page 24 |
| [**Table S15.** 61 valid instrumental variables used for Mendelian randomization analysis of mouth ulcers (Exposure) on insomnia (Outcome).](#S15) | Page 26 |
| [**Table S16.** 54 valid instrumental variables used for Mendelian randomization analysis of mouth ulcers (Exposure) on major depressive disorder (Outcome).](#S16) | Page 28 |
| [**Table S17.** 54 valid instrumental variables used for Mendelian randomization analysis of mouth ulcers (Exposure) on neuroticism (Outcome).](#S17) | Page 30 |
| [**Table S18.** 60 valid instrumental variables used for Mendelian randomization analysis of mouth ulcers (Exposure) on schizophrenia (Outcome).](#S18) | Page 32 |
| [**Table S19.** 28 valid instrumental variables used for Mendelian randomization analysis of mouth ulcers (Exposure) on subjective wellbeing (Outcome).](#S19) | Page 34 |
| [**Table S20.** Power calculation for two-sample MR analyses of psychiatric traits on mouth ulcers.](#S20) | Page 35 |
| [**Table S21.** Power calculation for two-sample MR analyses of mouth ulcers on psychiatric traits.](#S21) | Page 36 |
| [**Table S22.** Directional pleiotropy and heterogeneity test of the instrumental variables for psychiatric traits on mouth ulcers.](#S22) | Page 37 |
| [**Table S23.** Directional pleiotropy and heterogeneity test of the instrumental variables for mouth ulcers on psychiatric traits.](#S23) | Page 38 |

**Table S1. Description of GWAS phenotype for each trait.**

| **Trait** | **GWAS Phenotype Definition** |
| --- | --- |
| Anxiety disorders | Cases met one of two definitions. First was self-reporting a lifetime professional diagnosis of an anxiety disorder. Second was meeting criteria for a likely lifetime diagnosis of DSM-IV generalised anxiety disorder. |
| ADHD | Cases were diagnosed by psychiatrists at in- or out-patient clinics, predominantly the latter according to ICD10 (F90.0 diagnosis code). |
| ASD | For PGC sample, cases were those met from either the Autism Diagnostic Interview-Revised (ADI-R) or the Autism Diagnostic Observation Schedule (ADOS) domain scores. |
| BIP | Cases were required to meet international consensus criteria (DSM-IV, ICD-9, or ICD-10) for a lifetime diagnosis of BIP established using structured diagnostic instruments from assessments by trained  interviewers, clinician-administered checklists, or medical record review. |
| Insomnia | Sample whose answer is “usually” to question “Do you have trouble falling asleep at night or do you wake up in the middle of the night?” will be included as a case. |
| MDD | Cases were required to meet international consensus criteria (DSM-IV, ICD-9, or ICD-10) for a lifetime diagnosis of MDD established using structured diagnostic instruments from assessments by trained interviewers, clinician administered checklists, or medical record review. |
| Mood instability | Sample whose answer is “yes” to question “Does your mood often go up and down?” will be included as a case. |
| Neuroticism | A scale with 12 items measures the degree of neuroticism. |
| SCZ | Cases validation acquired both clinical and consensus research diagnosis. Details of cases validation is given in supplementary materials (PMID: 29483656). |
| Subjective wellbeing | A question of “How satisfied are you with your life?” with five options to choose (“Very dissatisfied”, “Somewhat dissatisfied”, “Neither dissatisfied nor satisfied”, “Somewhat satisfied”, “Very satisfied”). |

ADHD, attention deficit/hyperactivity disorder; ASD, autism spectrum disorder; BIP, bipolar disorder; MDD, major depressive disorder; SCZ, schizophrenia; for further description of all phenotypes see the main text or supplementary materials of individual paper.

**Table S2. 4 valid instrumental variables used for Mendelian randomization analysis of anxiety disorder (Exposure) on mouth ulcers (Outcome).**

| **SNP** | **Effect allele** | **Non-effect allele** | **Effect allele frequency** | **Beta** | **SE** | ***P*** |
| --- | --- | --- | --- | --- | --- | --- |
| rs10959883 | T | C | 0.910 | 0.150 | 0.023 | 2.90×10^-11^ |
| rs1187280 | G | A | 0.880 | 0.130 | 0.022 | 6.60×10^-09^ |
| rs2861139 | C | T | 0.620 | 0.130 | 0.022 | 2.60×10^-09^ |
| rs3807866 | A | G | 0.480 | 0.120 | 0.022 | 4.80×10^-08^ |

**Table S3. 10 valid instrumental variables used for Mendelian randomization analysis of attention deficit/hyperactivity disorder (Exposure) on mouth ulcers (Outcome).**

| **SNP** | **Effect allele** | **Non-effect allele** | **Effect allele frequency** | | **Beta** | **SE** | ***P*** |
| --- | --- | --- | --- | --- | --- | --- | --- |
| rs112361411 | C | T | - | 0.105 | | 0.015 | 1.13×10^-12^ |
| rs1222064 | T | C | - | 0.077 | | 0.016 | 6.85×10^-07^ |
| rs9677504 | A | G | - | 0.113 | | 0.021 | 9.83×10^-08^ |
| rs4858241 | T | G | - | 0.082 | | 0.014 | 8.17×10^-09^ |
| rs16884473 | C | T | - | 0.068 | | 0.014 | 8.56×10^-07^ |
| rs4916723 | C | A | - | 0.078 | | 0.014 | 1.81×10^-08^ |
| rs10262192 | A | G | - | 0.074 | | 0.014 | 3.66×10^-08^ |
| rs11591402 | T | A | - | 0.092 | | 0.016 | 1.76×10^-08^ |
| rs1427829 | A | G | - | 0.082 | | 0.014 | 1.35×10^-09^ |
| rs281324 | C | T | - | 0.079 | | 0.014 | 6.68×10^-09^ |

**Table S4. 10 valid instrumental variables used for Mendelian randomization analysis of autism spectrum disorder (Exposure) on mouth ulcers (Outcome).**

| **SNP** | **Effect allele** | **Non-effect allele** | **Effect allele frequency** | | **Beta** | **SE** | ***P*** |
| --- | --- | --- | --- | --- | --- | --- | --- |
| rs10099100 | C | G | 0.331 | 0.084 | | 0.015 | 1.07×10^-08^ |
| rs223239 | T | G | 0.311 | 0.072 | | 0.003 | 8.82×10^-07^ |
| rs2388334 | G | A | 0.483 | 0.065 | | 0.009 | 3.34×10^-12^ |
| rs325506 | C | G | 0.423 | 0.057 | | 0.009 | 3.26×10^-11^ |
| rs11787216 | C | T | 0.636 | 0.058 | | 0.010 | 1.99×10^-09^ |
| rs1452075 | T | C | 0.721 | 0.061 | | 0.010 | 3.17×10^-09^ |
| rs1620977 | A | G | 0.260 | 0.056 | | 0.010 | 6.66×10^-09^ |
| rs10149470 | G | A | 0.513 | 0.049 | | 0.008 | 8.52×10^-09^ |
| rs16854048 | A | C | 0.858 | 0.069 | | 0.012 | 1.29×10^-08^ |
| rs910805 | G | A | 0.240 | 0.096 | | 0.016 | 2.04×10^-09^ |

**Table S5. 19 valid instrumental variables used for Mendelian randomization analysis of bipolar disorder (Exposure) on mouth ulcers (Outcome).**

| **SNP** | **Effect allele** | **Non-effect allele** | **Effect allele frequency** | | **Beta** | **SE** | ***P*** |
| --- | --- | --- | --- | --- | --- | --- | --- |
| rs7544145 | T | C | 0.812 | 0.082 | | 0.015 | 4.83×10^-08^ |
| rs9834970 | C | T | 0.491 | 0.077 | | 0.011 | 5.72×10^-12^ |
| rs2302417 | T | A | 0.510 | 0.073 | | 0.011 | 6.59×10^-11^ |
| rs3804640 | A | G | 0.533 | 0.063 | | 0.011 | 1.99×10^-08^ |
| rs11724116 | C | T | 0.840 | 0.085 | | 0.015 | 2.37×10^-08^ |
| rs10035291 | T | C | 0.675 | 0.068 | | 0.012 | 2.67×10^-08^ |
| rs2388334 | G | A | 0.481 | 0.065 | | 0.011 | 4.01×10^-09^ |
| rs10455979 | G | C | 0.466 | 0.062 | | 0.011 | 4.31×10^-08^ |
| rs113779084 | A | G | 0.304 | 0.073 | | 0.012 | 2.51×10^-09^ |
| rs73188321 | C | T | 0.667 | 0.078 | | 0.013 | 1.06×10^-09^ |
| rs10994318 | C | G | 0.057 | 0.135 | | 0.023 | 6.76×10^-09^ |
| rs10896090 | A | G | 0.809 | 0.080 | | 0.014 | 1.90×10^-08^ |
| rs7122539 | G | A | 0.652 | 0.065 | | 0.012 | 3.77×10^-08^ |
| rs12575685 | A | G | 0.314 | 0.070 | | 0.012 | 7.71×10^-09^ |
| rs10744560 | T | C | 0.336 | 0.073 | | 0.012 | 3.62×10^-10^ |
| rs4447398 | A | C | 0.123 | 0.094 | | 0.016 | 9.37×10^-09^ |
| rs11647445 | G | T | 0.346 | 0.076 | | 0.012 | 1.08×10^-10^ |
| rs11557713 | A | G | 0.285 | 0.067 | | 0.012 | 3.64×10^-08^ |
| rs111444407 | T | C | 0.148 | 0.093 | | 0.015 | 1.33×10^-09^ |

**Table S6. 202 valid instrumental variables used for Mendelian randomization analysis of insomnia (Exposure) on mouth ulcers (Outcome).**

| **SNP** | **Effect allele** | **Non-effect allele** | **Effect allele frequency** | **Beta** | **SE** | ***P*** |
| --- | --- | --- | --- | --- | --- | --- |
| rs1064939 | A | T | 0.978 | 0.130 | 0.020 | 2.16×10^-10^ |
| rs79204944 | A | G | 0.045 | 0.079 | 0.014 | 4.24×10^-08^ |
| rs72899452 | T | C | 0.065 | 0.074 | 0.012 | 1.00×10^-09^ |
| rs55972276 | A | C | 0.137 | 0.073 | 0.009 | 4.19×10^-17^ |
| rs77641763 | T | C | 0.122 | 0.071 | 0.009 | 6.53×10^-15^ |
| rs138014720 | A | T | 0.941 | 0.070 | 0.013 | 3.46×10^-08^ |
| rs2286729 | A | G | 0.086 | 0.070 | 0.011 | 5.37×10^-11^ |
| rs118166957 | T | C | 0.159 | 0.068 | 0.008 | 1.95×10^-16^ |
| rs11650304 | C | G | 0.931 | 0.067 | 0.012 | 1.23×10^-08^ |
| rs62264767 | A | C | 0.853 | 0.065 | 0.008 | 1.63×10^-14^ |
| rs7168238 | C | G | 0.074 | 0.064 | 0.011 | 1.80×10^-08^ |
| rs699844 | A | G | 0.920 | 0.060 | 0.011 | 4.11×10^-08^ |
| rs28611339 | T | G | 0.128 | 0.058 | 0.009 | 8.46×10^-11^ |
| rs6465151 | T | C | 0.113 | 0.056 | 0.009 | 1.90×10^-09^ |
| rs16903122 | T | C | 0.249 | 0.055 | 0.007 | 9.04×10^-16^ |
| rs2792990 | C | G | 0.855 | 0.054 | 0.008 | 1.15×10^-10^ |
| rs34490907 | C | G | 0.888 | 0.054 | 0.009 | 1.76×10^-08^ |
| rs670501 | T | C | 0.213 | 0.053 | 0.007 | 7.40×10^-13^ |
| rs1927902 | T | C | 0.254 | 0.053 | 0.007 | 1.15×10^-14^ |
| rs1620977 | A | G | 0.270 | 0.052 | 0.007 | 2.27×10^-14^ |
| rs75452188 | A | G | 0.878 | 0.052 | 0.009 | 1.58×10^-08^ |
| rs11756035 | C | G | 0.128 | 0.051 | 0.009 | 1.29×10^-08^ |
| rs62429521 | A | C | 0.146 | 0.051 | 0.008 | 1.78×10^-09^ |
| rs7992992 | A | G | 0.129 | 0.051 | 0.009 | 1.15×10^-08^ |
| rs908668 | T | C | 0.208 | 0.050 | 0.007 | 1.41×10^-11^ |
| rs2491124 | T | C | 0.576 | 0.049 | 0.006 | 8.81×10^-16^ |
| rs35322724 | A | C | 0.577 | 0.049 | 0.006 | 3.75×10^-16^ |
| rs62068188 | T | C | 0.834 | 0.049 | 0.008 | 1.18×10^-09^ |
| rs3902952 | T | C | 0.188 | 0.048 | 0.008 | 2.55×10^-10^ |
| rs45453598 | A | T | 0.169 | 0.047 | 0.008 | 4.42×10^-09^ |
| rs17223714 | A | G | 0.789 | 0.046 | 0.007 | 2.44×10^-10^ |
| rs9316619 | T | C | 0.825 | 0.046 | 0.008 | 5.50×10^-09^ |
| rs429358 | T | C | 0.846 | 0.046 | 0.008 | 2.13×10^-08^ |
| rs12310246 | A | G | 0.249 | 0.045 | 0.007 | 4.74×10^-11^ |
| rs67501351 | C | G | 0.745 | 0.045 | 0.007 | 5.36×10^-11^ |
| rs34214423 | A | C | 0.809 | 0.045 | 0.008 | 3.18×10^-09^ |
| rs12614369 | A | G | 0.816 | 0.044 | 0.008 | 7.21×10^-09^ |
| rs116466468 | T | C | 0.759 | 0.044 | 0.007 | 2.11×10^-10^ |
| rs2903385 | A | G | 0.484 | 0.043 | 0.006 | 4.53×10^-13^ |
| rs6606731 | A | T | 0.192 | 0.043 | 0.008 | 1.51×10^-08^ |
| rs742760 | A | T | 0.816 | 0.043 | 0.008 | 2.48×10^-08^ |
| rs10800992 | T | C | 0.443 | 0.042 | 0.006 | 3.84×10^-12^ |
| rs55772859 | A | C | 0.311 | 0.042 | 0.006 | 4.82×10^-11^ |
| rs17005118 | A | G | 0.264 | 0.042 | 0.007 | 6.13×10^-10^ |
| rs12666306 | A | G | 0.502 | 0.042 | 0.006 | 2.24×10^-12^ |
| rs2867690 | T | C | 0.182 | 0.042 | 0.008 | 3.70×10^-08^ |
| rs17025198 | A | G | 0.204 | 0.041 | 0.007 | 2.19×10^-08^ |
| rs56133505 | A | G | 0.537 | 0.041 | 0.006 | 5.59×10^-12^ |
| rs715338 | A | G | 0.578 | 0.041 | 0.006 | 7.85×10^-12^ |
| rs4643373 | T | C | 0.701 | 0.041 | 0.007 | 1.58×10^-10^ |
| rs56097173 | T | C | 0.681 | 0.040 | 0.006 | 2.69×10^-10^ |
| rs12991815 | C | G | 0.424 | 0.040 | 0.006 | 3.02×10^-11^ |
| rs9373590 | A | T | 0.508 | 0.040 | 0.006 | 2.18×10^-11^ |
| rs4592425 | T | G | 0.697 | 0.040 | 0.006 | 4.31×10^-10^ |
| rs11149313 | A | G | 0.730 | 0.040 | 0.007 | 2.38×10^-09^ |
| rs6808140 | T | C | 0.505 | 0.039 | 0.006 | 5.35×10^-11^ |
| rs35110063 | A | G | 0.427 | 0.039 | 0.006 | 8.82×10^-11^ |
| rs1147852 | A | G | 0.310 | 0.039 | 0.006 | 9.94×10^-10^ |
| rs324017 | A | C | 0.294 | 0.039 | 0.007 | 1.61×10^-09^ |
| rs6562066 | T | C | 0.369 | 0.039 | 0.006 | 1.38×10^-10^ |
| rs1038093 | T | C | 0.628 | 0.039 | 0.006 | 2.47×10^-10^ |
| rs11090039 | A | G | 0.287 | 0.039 | 0.007 | 1.82×10^-09^ |
| rs1861412 | A | G | 0.434 | 0.038 | 0.006 | 1.67×10^-10^ |
| rs6888135 | A | C | 0.497 | 0.038 | 0.006 | 1.21×10^-10^ |
| rs940780 | T | C | 0.359 | 0.038 | 0.006 | 8.50×10^-10^ |
| rs12924275 | T | C | 0.268 | 0.038 | 0.007 | 1.93×10^-08^ |
| rs2398144 | A | C | 0.395 | 0.038 | 0.006 | 5.09×10^-10^ |
| rs11679943 | A | G | 0.347 | 0.037 | 0.006 | 3.16×10^-09^ |
| rs6756610 | C | G | 0.629 | 0.037 | 0.006 | 1.14×10^-09^ |
| rs62213452 | T | G | 0.279 | 0.037 | 0.007 | 2.39×10^-08^ |
| rs7040224 | A | G | 0.316 | 0.037 | 0.006 | 4.24×10^-09^ |
| rs72773790 | T | C | 0.673 | 0.037 | 0.006 | 3.71×10^-09^ |
| rs1530938 | A | G | 0.442 | 0.036 | 0.006 | 8.82×10^-10^ |
| rs7625896 | A | G | 0.655 | 0.036 | 0.006 | 5.28×10^-09^ |
| rs2737240 | A | G | 0.708 | 0.036 | 0.007 | 3.37×10^-08^ |
| rs10756571 | T | C | 0.685 | 0.036 | 0.006 | 1.80×10^-08^ |
| rs2221119 | C | G | 0.443 | 0.036 | 0.006 | 2.00×10^-09^ |
| rs9540729 | A | T | 0.479 | 0.036 | 0.006 | 1.40×10^-09^ |
| rs34967082 | A | G | 0.414 | 0.035 | 0.006 | 4.34×10^-09^ |
| rs2216427 | C | G | 0.653 | 0.035 | 0.006 | 1.60×10^-08^ |
| rs6601080 | A | G | 0.676 | 0.035 | 0.006 | 2.21×10^-08^ |
| rs2598293 | T | C | 0.476 | 0.035 | 0.006 | 2.48×10^-09^ |
| rs871994 | A | C | 0.435 | 0.035 | 0.006 | 5.50×10^-09^ |
| rs1167132 | T | C | 0.392 | 0.035 | 0.006 | 8.73×10^-09^ |
| rs176644 | T | G | 0.404 | 0.035 | 0.006 | 9.49×10^-09^ |
| rs12605642 | T | G | 0.486 | 0.035 | 0.006 | 2.13×10^-09^ |
| rs9964420 | A | C | 0.301 | 0.035 | 0.007 | 4.54×10^-08^ |
| rs72820274 | A | G | 0.417 | 0.034 | 0.006 | 1.28×10^-08^ |
| rs10928256 | T | C | 0.419 | 0.034 | 0.006 | 1.61×10^-08^ |
| rs4260410 | T | C | 0.332 | 0.034 | 0.006 | 4.87×10^-08^ |
| rs11722569 | T | C | 0.659 | 0.034 | 0.006 | 2.91×10^-08^ |
| rs13138995 | A | G | 0.390 | 0.034 | 0.006 | 1.97×10^-08^ |
| rs2030672 | C | G | 0.559 | 0.034 | 0.006 | 1.10×10^-08^ |
| rs10898940 | A | C | 0.517 | 0.034 | 0.006 | 8.09×10^-09^ |
| rs1567084 | A | G | 0.498 | 0.033 | 0.006 | 2.14×10^-08^ |
| rs1580173 | A | G | 0.561 | 0.033 | 0.006 | 2.28×10^-08^ |
| rs1357685 | T | C | 0.474 | 0.033 | 0.006 | 1.39×10^-08^ |
| rs4588900 | A | G | 0.516 | 0.033 | 0.006 | 1.57×10^-08^ |
| rs28552587 | A | G | 0.564 | 0.033 | 0.006 | 3.30×10^-08^ |
| rs10955647 | T | G | 0.532 | 0.033 | 0.006 | 1.84×10^-08^ |
| rs6597649 | T | C | 0.399 | 0.033 | 0.006 | 3.05×10^-08^ |
| rs10825503 | T | G | 0.487 | 0.033 | 0.006 | 1.43×10^-08^ |
| rs667730 | T | C | 0.579 | 0.033 | 0.006 | 2.26×10^-08^ |
| rs647905 | T | C | 0.541 | 0.033 | 0.006 | 2.87×10^-08^ |
| rs10947987 | C | T | 0.557 | 0.033 | 0.006 | 4.08×10^-08^ |
| rs4858708 | T | A | 0.470 | 0.034 | 0.006 | 1.23×10^-08^ |
| rs2364921 | C | T | 0.531 | 0.034 | 0.006 | 2.13×10^-08^ |
| rs190073 | G | A | 0.586 | 0.034 | 0.006 | 2.86×10^-08^ |
| rs9563886 | C | T | 0.394 | 0.034 | 0.006 | 3.08×10^-08^ |
| rs1553754 | G | T | 0.438 | 0.034 | 0.006 | 3.51×10^-08^ |
| rs11588755 | G | A | 0.478 | 0.035 | 0.006 | 5.14×10^-09^ |
| rs11119409 | C | T | 0.413 | 0.035 | 0.006 | 1.19×10^-08^ |
| rs728017 | G | A | 0.614 | 0.035 | 0.006 | 9.51×10^-09^ |
| rs1731951 | T | A | 0.557 | 0.035 | 0.006 | 1.36×10^-08^ |
| rs4788203 | G | A | 0.567 | 0.035 | 0.006 | 6.32×10^-09^ |
| rs12454003 | G | C | 0.518 | 0.035 | 0.006 | 4.94×10^-09^ |
| rs910187 | G | A | 0.627 | 0.035 | 0.006 | 1.63×10^-08^ |
| rs12520974 | C | T | 0.515 | 0.036 | 0.006 | 1.69×10^-09^ |
| rs701394 | G | A | 0.362 | 0.036 | 0.006 | 6.83×10^-09^ |
| rs37445 | G | A | 0.610 | 0.036 | 0.006 | 4.88×10^-09^ |
| rs17367725 | C | T | 0.649 | 0.036 | 0.006 | 9.29×10^-09^ |
| rs10758593 | G | A | 0.601 | 0.036 | 0.006 | 4.90×10^-09^ |
| rs7402939 | C | T | 0.624 | 0.036 | 0.006 | 5.19×10^-09^ |
| rs2838787 | G | A | 0.608 | 0.036 | 0.006 | 7.65×10^-09^ |
| rs6702604 | G | A | 0.416 | 0.037 | 0.006 | 1.30×10^-09^ |
| rs823247 | C | T | 0.521 | 0.037 | 0.006 | 5.25×10^-10^ |
| rs1519102 | G | C | 0.311 | 0.037 | 0.006 | 1.90×10^-08^ |
| rs1064213 | G | A | 0.521 | 0.037 | 0.006 | 6.41×10^-10^ |
| rs7599697 | C | T | 0.642 | 0.037 | 0.006 | 5.00×10^-09^ |
| rs2388840 | G | A | 0.424 | 0.037 | 0.006 | 1.37×10^-09^ |
| rs7475916 | G | C | 0.647 | 0.037 | 0.006 | 6.70×10^-09^ |
| rs4767645 | G | T | 0.539 | 0.037 | 0.006 | 6.47×10^-10^ |
| rs6510033 | G | A | 0.275 | 0.037 | 0.007 | 4.66×10^-08^ |
| rs623025 | C | T | 0.745 | 0.038 | 0.007 | 3.16×10^-08^ |
| rs73163783 | C | T | 0.277 | 0.038 | 0.007 | 1.39×10^-08^ |
| rs10944696 | G | A | 0.702 | 0.038 | 0.007 | 7.99×10^-09^ |
| rs6973090 | G | A | 0.750 | 0.038 | 0.007 | 4.31×10^-08^ |
| rs671985 | G | A | 0.548 | 0.038 | 0.006 | 2.79×10^-10^ |
| rs11001276 | T | A | 0.260 | 0.038 | 0.007 | 2.52×10^-08^ |
| rs214934 | T | A | 0.688 | 0.038 | 0.006 | 3.16×10^-09^ |
| rs6589988 | G | A | 0.324 | 0.038 | 0.006 | 4.70×10^-09^ |
| rs1536053 | C | T | 0.684 | 0.038 | 0.006 | 6.04×10^-09^ |
| rs3184470 | G | A | 0.649 | 0.038 | 0.006 | 9.73×10^-10^ |
| rs1937447 | G | C | 0.241 | 0.039 | 0.007 | 2.08×10^-08^ |
| rs7571486 | G | A | 0.749 | 0.039 | 0.007 | 1.40×10^-08^ |
| rs4502882 | C | T | 0.342 | 0.039 | 0.006 | 7.96×10^-10^ |
| rs4090240 | C | T | 0.723 | 0.039 | 0.007 | 8.46×10^-09^ |
| rs12251016 | T | A | 0.344 | 0.039 | 0.006 | 3.89×10^-10^ |
| rs224029 | C | T | 0.601 | 0.039 | 0.006 | 2.51×10^-10^ |
| rs566673 | G | T | 0.465 | 0.039 | 0.006 | 1.18×10^-10^ |
| rs7615602 | G | C | 0.729 | 0.040 | 0.007 | 2.59×10^-09^ |
| rs521484 | G | A | 0.233 | 0.040 | 0.007 | 1.53×10^-08^ |
| rs75932578 | C | T | 0.784 | 0.040 | 0.007 | 4.15×10^-08^ |
| rs12790660 | C | T | 0.316 | 0.040 | 0.006 | 4.49×10^-10^ |
| rs2389631 | C | A | 0.333 | 0.040 | 0.006 | 2.03×10^-10^ |
| rs2089358 | C | T | 0.296 | 0.041 | 0.007 | 2.75×10^-10^ |
| rs1289939 | C | T | 0.768 | 0.041 | 0.007 | 6.00×10^-09^ |
| rs11803128 | G | A | 0.346 | 0.041 | 0.006 | 6.85×10^-11^ |
| rs6545798 | T | A | 0.590 | 0.041 | 0.006 | 1.19×10^-11^ |
| rs4664299 | C | T | 0.765 | 0.041 | 0.007 | 4.95×10^-09^ |
| rs3774751 | G | T | 0.538 | 0.041 | 0.006 | 7.32×10^-12^ |
| rs7044885 | G | C | 0.558 | 0.041 | 0.006 | 5.67×10^-12^ |
| rs6734957 | G | T | 0.761 | 0.042 | 0.007 | 1.82×10^-09^ |
| rs62301574 | G | C | 0.200 | 0.042 | 0.007 | 1.37×10^-08^ |
| rs12917449 | C | A | 0.194 | 0.042 | 0.008 | 2.97×10^-08^ |
| rs9889282 | C | A | 0.387 | 0.042 | 0.006 | 4.70×10^-12^ |
| rs10761240 | G | A | 0.604 | 0.043 | 0.006 | 2.12×10^-12^ |
| rs12912299 | C | T | 0.511 | 0.043 | 0.006 | 4.42×10^-13^ |
| rs4238755 | C | A | 0.736 | 0.043 | 0.007 | 2.30×10^-10^ |
| rs12983032 | G | A | 0.657 | 0.043 | 0.006 | 1.07×10^-11^ |
| rs694786 | C | T | 0.540 | 0.044 | 0.006 | 1.97×10^-13^ |
| rs17083297 | C | A | 0.823 | 0.044 | 0.008 | 1.60×10^-08^ |
| rs6967168 | G | T | 0.246 | 0.044 | 0.007 | 1.39×10^-10^ |
| rs524859 | G | A | 0.640 | 0.044 | 0.006 | 1.48×10^-12^ |
| rs61921611 | C | T | 0.308 | 0.044 | 0.006 | 7.84×10^-12^ |
| rs7214267 | G | A | 0.419 | 0.044 | 0.006 | 5.09×10^-13^ |
| rs61765555 | C | T | 0.745 | 0.045 | 0.007 | 4.00×10^-11^ |
| rs11605348 | G | A | 0.651 | 0.045 | 0.006 | 7.01×10^-13^ |
| rs16990210 | C | T | 0.152 | 0.046 | 0.008 | 1.97×10^-08^ |
| rs12540241 | T | A | 0.807 | 0.046 | 0.008 | 1.58×10^-09^ |
| rs10947690 | G | A | 0.259 | 0.047 | 0.007 | 4.04×10^-12^ |
| rs4702 | G | A | 0.444 | 0.048 | 0.006 | 6.78×10^-16^ |
| rs73079014 | C | T | 0.874 | 0.049 | 0.009 | 3.65×10^-08^ |
| rs8180817 | G | C | 0.570 | 0.049 | 0.006 | 1.83×10^-16^ |
| rs76145129 | G | T | 0.876 | 0.050 | 0.009 | 2.73×10^-08^ |
| rs1031654 | C | A | 0.200 | 0.051 | 0.007 | 3.88×10^-12^ |
| rs152555 | G | A | 0.146 | 0.052 | 0.008 | 4.83×10^-10^ |
| rs2431108 | C | T | 0.328 | 0.053 | 0.006 | 7.83×10^-17^ |
| rs9394502 | C | T | 0.666 | 0.054 | 0.006 | 7.76×10^-18^ |
| rs4709655 | C | T | 0.881 | 0.054 | 0.009 | 3.09×10^-09^ |
| rs28582096 | G | A | 0.795 | 0.054 | 0.007 | 1.74×10^-13^ |
| rs4981170 | G | A | 0.806 | 0.054 | 0.008 | 7.33×10^-13^ |
| rs72657797 | C | T | 0.824 | 0.056 | 0.008 | 1.52×10^-12^ |
| rs8180457 | C | T | 0.843 | 0.056 | 0.008 | 1.12×10^-11^ |
| rs73671843 | G | A | 0.874 | 0.056 | 0.009 | 5.49×10^-10^ |
| rs13010288 | G | T | 0.867 | 0.060 | 0.009 | 9.26×10^-12^ |
| rs62383308 | G | A | 0.920 | 0.060 | 0.011 | 3.98×10^-08^ |
| rs17643634 | C | T | 0.835 | 0.060 | 0.008 | 1.34×10^-13^ |
| rs492858 | C | T | 0.924 | 0.066 | 0.011 | 3.46×10^-09^ |
| rs7432782 | C | T | 0.044 | 0.083 | 0.014 | 7.42×10^-09^ |
| rs13135092 | G | A | 0.083 | 0.089 | 0.011 | 2.53×10^-16^ |
| rs117630493 | G | C | 0.027 | 0.101 | 0.018 | 3.61×10^-08^ |

**Table S7. 28 valid instrumental variables used for Mendelian randomization analysis of major depressive disorder (Exposure) on mouth ulcers (Outcome).**

| **SNP** | **Effect allele** | **Non-effect allele** | **Effect allele frequency** | | **Beta** | **SE** | ***P*** |
| --- | --- | --- | --- | --- | --- | --- | --- |
| rs1432639 | A | C | 0.630 | 0.039 | | 0.005 | 4.60×10^-15^ |
| rs12129573 | A | C | 0.370 | 0.039 | | 0.005 | 4.00×10^-12^ |
| rs2389016 | T | C | 0.280 | 0.030 | | 0.005 | 1.00×10^-08^ |
| rs4261101 | G | A | 0.630 | 0.030 | | 0.005 | 1.00×10^-08^ |
| rs1226412 | T | C | 0.790 | 0.030 | | 0.006 | 2.40×10^-08^ |
| rs76308640^$^ | G | A | 0.098 | 0.038 | | 0.013 | 0.0039 |
| rs7430565 | G | A | 0.420 | 0.030 | | 0.005 | 2.90×10^-09^ |
| rs34215985 | G | C | 0.760 | 0.041 | | 0.006 | 3.10×10^-09^ |
| rs27732^$^ | A | G | 0.412 | 0.027 | | 0.008 | 0.0007 |
| rs2018142 | C | A | 0.470 | 0.048 | | 0.008 | 8.36×10^-10^ |
| rs11135349 | C | A | 0.520 | 0.030 | | 0.005 | 1.10×10^-09^ |
| rs9402472 | A | G | 0.240 | 0.030 | | 0.006 | 2.80×10^-08^ |
| rs12666117 | A | G | 0.470 | 0.030 | | 0.005 | 1.40×10^-08^ |
| rs7856424 | C | T | 0.710 | 0.030 | | 0.005 | 8.50×10^-09^ |
| rs7029033 | T | C | 0.070 | 0.049 | | 0.009 | 2.70×10^-08^ |
| rs61867293 | C | T | 0.800 | 0.041 | | 0.006 | 7.00×10^-10^ |
| rs1806153 | T | G | 0.220 | 0.039 | | 0.006 | 1.20×10^-09^ |
| rs4074723 | C | A | 0.590 | 0.030 | | 0.005 | 3.10×10^-08^ |
| rs12552 | A | G | 0.440 | 0.039 | | 0.005 | 6.10×10^-19^ |
| rs4904738 | C | T | 0.430 | 0.030 | | 0.005 | 2.60×10^-09^ |
| rs915057 | G | A | 0.580 | 0.030 | | 0.005 | 7.60×10^-10^ |
| rs10149470 | G | A | 0.510 | 0.030 | | 0.005 | 3.10×10^-09^ |
| rs8025231 | C | A | 0.430 | 0.030 | | 0.005 | 2.40×10^-12^ |
| rs8063603 | G | A | 0.350 | 0.030 | | 0.005 | 6.90×10^-09^ |
| rs7198928 | T | C | 0.620 | 0.030 | | 0.005 | 1.00×10^-08^ |
| rs7200826 | T | C | 0.250 | 0.030 | | 0.006 | 2.40×10^-08^ |
| rs17727765 | C | T | 0.080 | 0.051 | | 0.009 | 8.50×10^-09^ |
| rs5758265 | A | G | 0.280 | 0.030 | | 0.005 | 7.60×10^-09^ |

$, SNP could not be found in summary statistics dataset of mouth ulcers, and the proxy SNP (r^2^ > 0.6) were selected subsequently.

**Table S8. 26 valid instrumental variables used for Mendelian randomization analysis of mood instability (Exposure) on mouth ulcers (Outcome).**

| **SNP** | **Effect allele** | **Non-effect allele** | | **Effect allele frequency** | | **Beta** | **SE** | ***P*** |
| --- | --- | --- | --- | --- | --- | --- | --- | --- |
| rs2000228 | C | T | - | | 0.007 | | 0.001 | 6.50×10^-09^ |
| rs12477961 | A | G | - | | 0.006 | | 0.001 | 3.30×10^-08^ |
| rs12486699 | A | C | - | | 0.007 | | 0.001 | 4.80×10^-08^ |
| rs73082357 | G | A | - | | 0.012 | | 0.002 | 4.20×10^-11^ |
| rs4082244 | C | G | - | | 0.007 | | 0.001 | 2.20×10^-08^ |
| rs45510500 | C | T | - | | 0.014 | | 0.002 | 3.30×10^-08^ |
| rs6882578 | C | G | - | | 0.006 | | 0.001 | 2.10×10^-08^ |
| rs6861117 | A | G | - | | 0.012 | | 0.002 | 1.80×10^-08^ |
| rs67447472 | T | G | - | | 0.011 | | 0.002 | 3.80×10^-09^ |
| rs2483509 | A | G | - | | 0.006 | | 0.001 | 2.00×10^-08^ |
| rs7818437 | C | T | - | | 0.010 | | 0.001 | 2.60×10^-13^ |
| rs1962104 | C | T | - | | 0.008 | | 0.001 | 7.70×10^-12^ |
| rs56116032 | A | G | - | | 0.009 | | 0.001 | 6.10×10^-11^ |
| rs9775606 | C | G | - | | 0.007 | | 0.001 | 1.10×10^-08^ |
| rs999483 | G | T | - | | 0.008 | | 0.001 | 1.90×10^-10^ |
| rs11599236 | T | C | - | | 0.007 | | 0.001 | 5.40×10^-10^ |
| rs16932966 | A | G | - | | 0.008 | | 0.001 | 3.10×10^-10^ |
| rs72995548 | C | T | - | | 0.014 | | 0.002 | 3.20×10^-09^ |
| rs61915924 | A | C | - | | 0.008 | | 0.001 | 1.30×10^-08^ |
| rs8045174 | T | C | - | | 0.009 | | 0.001 | 1.00×10^-09^ |
| rs1559422 | C | T | - | | 0.007 | | 0.001 | 3.20×10^-08^ |
| rs1050863 | G | A | - | | 0.008 | | 0.001 | 2.60×10^-12^ |
| rs4799949 | C | T | - | | 0.008 | | 0.001 | 6.50×10^-10^ |
| rs56403421 | C | A | - | | 0.008 | | 0.001 | 3.10×10^-11^ |
| rs613872 | T | G | - | | 0.012 | | 0.002 | 9.90×10^-15^ |
| rs6103271 | A | G | - | | 0.009 | | 0.002 | 6.40×10^-09^ |

**Table S9. 29 valid instrumental variables used for Mendelian randomization analysis of neuroticism (Exposure) on mouth ulcers (Outcome).**

| **SNP** | **Effect allele** | **Non-effect allele** | | **Effect allele frequency** | | **Beta** | **SE** | ***P*** |
| --- | --- | --- | --- | --- | --- | --- | --- | --- |
| rs8084351 | A | G | 0.509 | | 0.021 | | 0.003 | 1.47×10^-13^ |
| rs4938021 | T | C | 0.620 | | 0.021 | | 0.003 | 7.86×10^-13^ |
| rs11082011 | C | T | 0.334 | | 0.021 | | 0.003 | 2.99×10^-12^ |
| rs7218235 | A | G | 0.208 | | 0.024 | | 0.004 | 1.41×10^-11^ |
| rs10809520 | C | T | 0.717 | | 0.021 | | 0.003 | 1.94×10^-11^ |
| rs2717036 | C | T | 0.599 | | 0.019 | | 0.003 | 6.11×10^-11^ |
| rs2451500 | C | T | 0.347 | | 0.019 | | 0.003 | 6.23×10^-11^ |
| rs4257287 | T | C | 0.886 | | 0.029 | | 0.004 | 8.05×10^-11^ |
| rs35991856 | A | C | 0.133 | | 0.027 | | 0.004 | 8.42×10^-11^ |
| rs17828731 | T | C | 0.555 | | 0.018 | | 0.003 | 2.87×10^-10^ |
| rs589249 | G | A | 0.309 | | 0.019 | | 0.003 | 3.17×10^-10^ |
| rs10244364 | C | T | 0.314 | | 0.019 | | 0.003 | 7.35×10^-10^ |
| rs171697 | G | C | 0.331 | | 0.018 | | 0.003 | 1.82×10^-09^ |
| rs2149351 | T | G | 0.242 | | 0.019 | | 0.003 | 2.68×10^-09^ |
| rs1690816 | C | T | 0.313 | | 0.018 | | 0.003 | 2.98×10^-09^ |
| rs2273085 | T | C | 0.291 | | 0.018 | | 0.003 | 3.62×10^-09^ |
| rs1400867 | G | A | 0.878 | | 0.025 | | 0.004 | 4.59×10^-09^ |
| rs2359239 | C | T | 0.614 | | 0.017 | | 0.003 | 5.59×10^-09^ |
| rs7107356 | G | A | 0.507 | | 0.016 | | 0.003 | 6.53×10^-09^ |
| rs1806153 | T | G | 0.227 | | 0.020 | | 0.003 | 8.77×10^-09^ |
| rs12903563 | T | C | 0.482 | | 0.016 | | 0.003 | 1.04×10^-08^ |
| rs10434704 | C | T | 0.533 | | 0.016 | | 0.003 | 1.16×10^-08^ |
| rs1111818 | G | C | 0.365 | | 0.017 | | 0.003 | 1.19×10^-08^ |
| rs2398144 | A | C | 0.394 | | 0.017 | | 0.003 | 1.26×10^-08^ |
| rs4918814 | A | C | 0.717 | | 0.018 | | 0.003 | 1.49×10^-08^ |
| rs703410 | G | A | 0.748 | | 0.018 | | 0.003 | 2.17×10^-08^ |
| rs4908768 | A | G | 0.194 | | 0.020 | | 0.004 | 2.19×10^-08^ |
| rs9944275 | T | A | 0.917 | | 0.028 | | 0.005 | 4.53×10^-08^ |
| rs11785693 | A | T | 0.793 | | 0.019 | | 0.003 | 4.92×10^-08^ |

**Table S10. 106 valid instrumental variables used for Mendelian randomization analysis of schizophrenia (Exposure) on mouth ulcers (Outcome).**

| **SNP** | **Effect allele** | **Non-effect allele** | **Effect allele frequency** | | **Beta** | **SE** | ***P*** |
| --- | --- | --- | --- | --- | --- | --- | --- |
| rs2007044 | G | A | 0.366 | 0.089 | | 0.010 | 5.63×10^-20^ |
| rs2660304 | T | G | 0.789 | 0.109 | | 0.012 | 2.18×10^-18^ |
| rs12416331 | T | A | 0.913 | 0.146 | | 0.017 | 7.09×10^-18^ |
| rs2949006 | T | G | 0.182 | 0.100 | | 0.012 | 3.69×10^-17^ |
| rs6065094 | G | A | 0.647 | 0.086 | | 0.010 | 7.91×10^-17^ |
| rs13107325 | T | C | 0.080 | 0.160 | | 0.019 | 1.19×10^-16^ |
| rs4144797 | T | C | 0.386 | 0.082 | | 0.010 | 4.33×10^-16^ |
| rs2851447 | G | C | 0.276 | 0.087 | | 0.011 | 5.55×10^-16^ |
| rs12293670 | A | G | 0.649 | 0.081 | | 0.010 | 1.70×10^-15^ |
| rs12129573 | A | C | 0.397 | 0.077 | | 0.010 | 8.94×10^-15^ |
| rs6002655 | T | C | 0.425 | 0.075 | | 0.010 | 2.15×10^-14^ |
| rs4766428 | T | C | 0.453 | 0.076 | | 0.010 | 2.68×10^-14^ |
| rs10083370 | G | A | 0.383 | 0.075 | | 0.010 | 3.44×10^-14^ |
| rs12704290 | G | A | 0.872 | 0.114 | | 0.015 | 3.57×10^-14^ |
| rs7701440 | C | T | 0.474 | 0.073 | | 0.010 | 3.72×10^-14^ |
| rs4936215 | A | G | 0.779 | 0.092 | | 0.012 | 5.32×10^-14^ |
| rs7951870 | C | T | 0.176 | 0.093 | | 0.013 | 2.99×10^-13^ |
| rs6434928 | G | A | 0.274 | 0.073 | | 0.010 | 3.62×10^-13^ |
| rs7893279 | T | G | 0.893 | 0.112 | | 0.015 | 4.80×10^-13^ |
| rs11646127 | G | C | 0.556 | 0.070 | | 0.010 | 5.52×10^-13^ |
| rs9607782 | A | T | 0.265 | 0.081 | | 0.011 | 5.54×10^-13^ |
| rs217287 | C | T | 0.556 | 0.070 | | 0.010 | 9.53×10^-13^ |
| rs5757730 | G | A | 0.563 | 0.071 | | 0.010 | 1.76×10^-12^ |
| rs7596038 | C | T | 0.565 | 0.068 | | 0.010 | 2.37×10^-12^ |
| rs35346733 | C | T | 0.188 | 0.072 | | 0.010 | 2.42×10^-12^ |
| rs2514218 | A | G | 0.648 | 0.087 | | 0.012 | 2.42×10^-12^ |
| rs17514846 | C | A | 0.539 | 0.068 | | 0.010 | 2.55×10^-12^ |
| rs1080500 | G | A | 0.666 | 0.072 | | 0.010 | 2.71×10^-12^ |
| rs3743078 | G | C | 0.756 | 0.078 | | 0.011 | 3.11×10^-12^ |
| rs34796896 | G | A | 0.793 | 0.083 | | 0.012 | 3.19×10^-12^ |
| rs7432375 | A | G | 0.565 | 0.080 | | 0.012 | 4.07×10^-12^ |
| rs36043959 | G | A | 0.192 | 0.068 | | 0.010 | 4.07×10^-12^ |
| rs1451488 | G | A | 0.526 | 0.067 | | 0.010 | 4.75×10^-12^ |
| rs6694545 | A | G | 0.239 | 0.077 | | 0.011 | 6.20×10^-12^ |
| rs2905432 | G | A | 0.332 | 0.069 | | 0.010 | 6.62×10^-12^ |
| rs4648845 | T | C | 0.476 | 0.077 | | 0.011 | 6.74×10^-12^ |
| rs11783093 | C | T | 0.846 | 0.093 | | 0.014 | 7.64×10^-12^ |
| rs111294930 | A | G | 0.719 | 0.083 | | 0.012 | 9.04×10^-12^ |
| rs62334820 | T | C | 0.192 | 0.081 | | 0.012 | 9.60×10^-12^ |
| rs2970610 | T | C | 0.362 | 0.068 | | 0.010 | 1.39×10^-11^ |
| rs16867576 | A | G | 0.890 | 0.101 | | 0.015 | 1.65×10^-11^ |
| rs1473594 | T | C | 0.397 | 0.064 | | 0.010 | 3.33×10^-11^ |
| rs16902086 | G | A | 0.358 | 0.066 | | 0.010 | 5.55×10^-11^ |
| rs3735025 | T | C | 0.640 | 0.065 | | 0.010 | 7.02×10^-11^ |
| rs1319017 | A | G | 0.354 | 0.066 | | 0.010 | 7.82×10^-11^ |
| rs75968099 | T | C | 0.363 | 0.066 | | 0.010 | 9.41×10^-11^ |
| rs704373 | A | G | 0.356 | 0.065 | | 0.010 | 1.39×10^-10^ |
| rs2332700 | C | G | 0.247 | 0.070 | | 0.011 | 1.52×10^-10^ |
| rs14403 | C | T | 0.793 | 0.074 | | 0.012 | 1.71×10^-10^ |
| rs2053079 | G | A | 0.259 | 0.070 | | 0.011 | 1.82×10^-10^ |
| rs42945 | G | A | 0.558 | 0.063 | | 0.010 | 2.25×10^-10^ |
| rs2917569 | T | C | 0.499 | 0.061 | | 0.010 | 3.11×10^-10^ |
| rs1191551 | T | G | 0.225 | 0.073 | | 0.012 | 4.12×10^-10^ |
| rs7499750 | A | C | 0.260 | 0.070 | | 0.011 | 4.24×10^-10^ |
| rs72769124 | A | C | 0.096 | 0.109 | | 0.017 | 4.73×10^-10^ |
| rs10148671 | C | T | 0.631 | 0.063 | | 0.010 | 5.46×10^-10^ |
| rs10156310 | A | T | 0.758 | 0.073 | | 0.012 | 5.56×10^-10^ |
| rs12991836 | C | A | 0.372 | 0.061 | | 0.010 | 6.46×10^-10^ |
| rs140505938 | C | T | 0.831 | 0.083 | | 0.014 | 6.50×10^-10^ |
| rs13169274 | C | T | 0.500 | 0.060 | | 0.010 | 7.06×10^-10^ |
| rs72986630 | T | C | 0.059 | 0.140 | | 0.023 | 8.09×10^-10^ |
| rs783540 | G | A | 0.419 | 0.059 | | 0.010 | 8.45×10^-10^ |
| rs12908161 | A | G | 0.736 | 0.067 | | 0.011 | 9.41×10^-10^ |
| rs10985817 | C | T | 0.166 | 0.080 | | 0.013 | 1.02×10^-09^ |
| rs1899543 | T | A | 0.552 | 0.058 | | 0.009 | 1.23×10^-09^ |
| rs56775891 | T | C | 0.250 | 0.066 | | 0.011 | 2.03×10^-09^ |
| rs211829 | T | C | 0.577 | 0.059 | | 0.010 | 2.29×10^-09^ |
| rs12898315 | A | G | 0.568 | 0.057 | | 0.010 | 2.51×10^-09^ |
| rs1042992 | T | C | 0.165 | 0.074 | | 0.012 | 3.67×10^-09^ |
| rs17465671 | C | A | 0.492 | 0.057 | | 0.010 | 4.14×10^-09^ |
| rs10196799 | A | T | 0.579 | 0.056 | | 0.010 | 4.51×10^-09^ |
| rs10783624 | C | A | 0.344 | 0.060 | | 0.010 | 5.44×10^-09^ |
| rs1353545 | C | G | 0.345 | 0.059 | | 0.010 | 5.67×10^-09^ |
| rs7801375 | G | A | 0.847 | 0.077 | | 0.013 | 6.27×10^-09^ |
| rs7191183 | C | T | 0.321 | 0.060 | | 0.010 | 6.31×10^-09^ |
| rs7010876 | T | A | 0.284 | 0.062 | | 0.011 | 6.51×10^-09^ |
| rs6035706 | G | A | 0.297 | 0.060 | | 0.010 | 7.24×10^-09^ |
| rs36104021 | C | G | 0.869 | 0.091 | | 0.016 | 7.31×10^-09^ |
| rs12712510 | T | C | 0.473 | 0.057 | | 0.010 | 8.18×10^-09^ |
| rs7632921 | G | T | 0.564 | 0.056 | | 0.010 | 9.52×10^-09^ |
| rs2410572 | G | A | 0.428 | 0.054 | | 0.010 | 1.07×10^-08^ |
| rs1765142 | A | C | 0.662 | 0.058 | | 0.010 | 1.13×10^-08^ |
| rs9545047 | A | C | 0.607 | 0.056 | | 0.010 | 1.15×10^-08^ |
| rs489939 | G | A | 0.631 | 0.057 | | 0.010 | 1.24×10^-08^ |
| rs634940 | T | G | 0.244 | 0.063 | | 0.011 | 1.30×10^-08^ |
| rs55669358 | C | T | 0.105 | 0.095 | | 0.017 | 1.37×10^-08^ |
| rs215411 | A | T | 0.305 | 0.058 | | 0.010 | 1.40×10^-08^ |
| rs1120004 | T | G | 0.244 | 0.062 | | 0.011 | 1.42×10^-08^ |
| rs12447542 | A | G | 0.143 | 0.086 | | 0.015 | 1.44×10^-08^ |
| rs760608 | G | A | 0.265 | 0.061 | | 0.011 | 1.90×10^-08^ |
| rs6800435 | A | C | 0.124 | 0.082 | | 0.015 | 2.00×10^-08^ |
| rs58950470 | T | G | 0.337 | 0.058 | | 0.010 | 2.07×10^-08^ |
| rs4240748 | G | C | 0.626 | 0.056 | | 0.010 | 2.15×10^-08^ |
| rs281299 | T | C | 0.615 | 0.056 | | 0.010 | 2.19×10^-08^ |
| rs56282503 | C | T | 0.304 | 0.062 | | 0.011 | 2.30×10^-08^ |
| rs6680011 | C | A | 0.147 | 0.074 | | 0.013 | 2.83×10^-08^ |
| rs2077586 | A | G | 0.728 | 0.061 | | 0.011 | 2.96×10^-08^ |
| rs893949 | C | T | 0.465 | 0.054 | | 0.010 | 2.98×10^-08^ |
| rs12129719 | A | G | 0.564 | 0.054 | | 0.010 | 3.35×10^-08^ |
| rs11993663 | A | C | 0.308 | 0.056 | | 0.010 | 3.40×10^-08^ |
| rs1975802 | G | A | 0.167 | 0.069 | | 0.013 | 3.56×10^-08^ |
| rs11165867 | T | C | 0.161 | 0.069 | | 0.013 | 3.87×10^-08^ |
| rs13121251 | T | C | 0.659 | 0.057 | | 0.010 | 4.06×10^-08^ |
| rs2161711 | A | G | 0.816 | 0.070 | | 0.013 | 4.22×10^-08^ |
| rs7225476 | A | G | 0.564 | 0.052 | | 0.010 | 4.86×10^-08^ |
| rs198160 | G | T | 0.501 | 0.056 | | 0.010 | 4.88×10^-08^ |

**Table S11. 34 valid instrumental variables used for Mendelian randomization analysis of subjective wellbeing (Exposure) on mouth ulcers (Outcome).**

| **SNP** | **Effect allele** | **Non-effect allele** | | **Effect allele frequency** | | **Beta** | **SE** | ***P*** |
| --- | --- | --- | --- | --- | --- | --- | --- | --- |
| rs1961639 | A | G | 0.663 | | 0.014 | | 0.002 | 2.26×10^-12^ |
| rs12187898 | T | C | 0.519 | | 0.013 | | 0.002 | 2.34×10^-12^ |
| rs148466862 | C | T | 0.959 | | 0.032 | | 0.005 | 7.99×10^-12^ |
| rs34761973 | C | T | 0.218 | | 0.015 | | 0.002 | 3.92×10^-11^ |
| rs12642606 | G | A | 0.504 | | 0.012 | | 0.002 | 5.70×10^-11^ |
| rs6063085 | C | A | 0.369 | | 0.013 | | 0.002 | 6.05×10^-11^ |
| rs10514301 | C | T | 0.876 | | 0.018 | | 0.003 | 9.64×10^-11^ |
| rs6579956 | G | T | 0.525 | | 0.012 | | 0.002 | 1.43×10^-10^ |
| rs4478137 | A | G | 0.630 | | 0.012 | | 0.002 | 2.90×10^-10^ |
| rs2075679 | C | T | 0.426 | | 0.012 | | 0.002 | 4.16×10^-10^ |
| rs1941687 | A | C | 0.598 | | 0.012 | | 0.002 | 4.74×10^-10^ |
| rs1400867 | A | G | 0.127 | | 0.017 | | 0.003 | 1.26×10^-09^ |
| rs172404 | A | C | 0.607 | | 0.011 | | 0.002 | 1.99×10^-09^ |
| rs993884 | T | C | 0.628 | | 0.011 | | 0.002 | 2.89×10^-09^ |
| rs11665070 | A | G | 0.669 | | 0.012 | | 0.002 | 3.23×10^-09^ |
| rs3824535 | G | T | 0.126 | | 0.017 | | 0.003 | 4.16×10^-09^ |
| rs228568 | G | A | 0.203 | | 0.013 | | 0.002 | 5.89×10^-09^ |
| rs716508 | T | C | 0.669 | | 0.012 | | 0.002 | 5.95×10^-09^ |
| rs4908768 | G | A | 0.802 | | 0.014 | | 0.002 | 6.28×10^-09^ |
| rs17820772 | T | C | 0.288 | | 0.012 | | 0.002 | 6.49×10^-09^ |
| rs531639 | T | C | 0.628 | | 0.011 | | 0.002 | 6.56×10^-09^ |
| rs4442212 | C | T | 0.560 | | 0.011 | | 0.002 | 6.85×10^-09^ |
| rs7617480 | C | A | 0.771 | | 0.013 | | 0.002 | 8.51×10^-09^ |
| rs11855468 | A | G | 0.952 | | 0.025 | | 0.004 | 1.20×10^-08^ |
| rs677325 | C | A | 0.868 | | 0.015 | | 0.003 | 1.46×10^-08^ |
| rs2398144 | C | A | 0.594 | | 0.011 | | 0.002 | 1.47×10^-08^ |
| rs6787782 | G | T | 0.859 | | 0.015 | | 0.003 | 2.00×10^-08^ |
| rs703410 | A | G | 0.263 | | 0.012 | | 0.002 | 2.20×10^-08^ |
| rs6759922 | G | A | 0.549 | | 0.010 | | 0.002 | 2.66×10^-08^ |
| rs35995292 | T | G | 0.681 | | 0.011 | | 0.002 | 2.83×10^-08^ |
| rs10498870 | A | G | 0.299 | | 0.011 | | 0.002 | 4.13×10^-08^ |
| rs2243616 | T | G | 0.646 | | 0.011 | | 0.002 | 4.47×10^-08^ |
| rs10434704 | T | C | 0.471 | | 0.010 | | 0.002 | 4.72×10^-08^ |
| rs10953620 | A | C | 0.522 | | 0.010 | | 0.002 | 4.81×10^-08^ |

**Table S12. 65 valid instrumental variables used for Mendelian randomization analysis of mouth ulcers (Exposure) on attention deficit/hyperactivity disorder (Outcome).**

| **SNP** | **Effect allele** | **Non-effect allele** | | **Effect allele frequency** | | **Beta** | **SE** | ***P*** |
| --- | --- | --- | --- | --- | --- | --- | --- | --- |
| rs11204668 | G | A | 0.436 | | 0.044 | | 0.007 | 3.00×10^-10^ |
| rs2759663 | C | G | 0.779 | | 0.045 | | 0.008 | 3.80×10^-08^ |
| rs3813961 | G | C | 0.015 | | 0.193 | | 0.029 | 3.60×10^-11^ |
| rs17015865 | G | A | 0.743 | | 0.048 | | 0.008 | 1.10×10^-09^ |
| rs4845140 | C | T | 0.957 | | 0.131 | | 0.017 | 2.20×10^-14^ |
| rs2232359 | G | A | 0.083 | | 0.101 | | 0.012 | 4.00×10^-16^ |
| rs35268627 | C | T | 0.124 | | 0.059 | | 0.010 | 1.80×10^-08^ |
| rs16823566 | G | T | 0.921 | | 0.087 | | 0.013 | 1.00×10^-11^ |
| rs11684030 | G | A | 0.368 | | 0.072 | | 0.007 | 3.00×10^-24^ |
| rs1491958 | T | C | 0.080 | | 0.086 | | 0.013 | 1.00×10^-11^ |
| rs12629300 | T | A | 0.174 | | 0.085 | | 0.009 | 1.00×10^-20^ |
| rs73156502 | T | C | 0.023 | | 0.131 | | 0.023 | 1.50×10^-08^ |
| rs145743887 | T | A | 0.014 | | 0.202 | | 0.030 | 3.20×10^-11^ |
| rs150921465 | T | G | 0.016 | | 0.214 | | 0.028 | 4.30×10^-14^ |
| rs116295688 | T | G | 0.025 | | 0.162 | | 0.024 | 7.70×10^-12^ |
| rs6805758 | A | C | 0.669 | | 0.065 | | 0.007 | 5.80×10^-19^ |
| rs7645203 | C | T | 0.595 | | 0.084 | | 0.007 | 4.20×10^-33^ |
| rs73170558 | C | T | 0.904 | | 0.087 | | 0.012 | 1.90×10^-13^ |
| rs668998 | A | G | 0.562 | | 0.071 | | 0.007 | 8.00×10^-25^ |
| rs4680564 | A | G | 0.609 | | 0.056 | | 0.007 | 1.90×10^-15^ |
| rs55667203 | T | C | 0.169 | | 0.097 | | 0.009 | 2.60×10^-25^ |
| rs145925847 | A | T | 0.012 | | 0.226 | | 0.032 | 2.10×10^-12^ |
| rs150383292 | T | C | 0.016 | | 0.212 | | 0.028 | 5.30×10^-14^ |
| rs4699030 | G | C | 0.582 | | 0.041 | | 0.007 | 3.10×10^-09^ |
| rs62351974 | C | T | 0.711 | | 0.044 | | 0.008 | 7.80×10^-09^ |
| rs4235479 | C | T | 0.501 | | 0.049 | | 0.007 | 8.10×10^-13^ |
| rs77630070 | G | T | 0.817 | | 0.052 | | 0.009 | 5.00×10^-09^ |
| rs4313034 | T | C | 0.817 | | 0.051 | | 0.009 | 1.10×10^-08^ |
| rs2517664 | C | T | 0.813 | | 0.059 | | 0.009 | 1.90×10^-11^ |
| rs78479381 | T | A | 0.098 | | 0.071 | | 0.012 | 7.10×10^-10^ |
| rs2074478 | T | C | 0.809 | | 0.053 | | 0.009 | 1.40×10^-09^ |
| rs2523589 | T | G | 0.496 | | 0.073 | | 0.007 | 2.70×10^-26^ |
| rs76518703 | G | A | 0.052 | | 0.129 | | 0.016 | 1.50×10^-15^ |
| rs138753323 | C | T | 0.033 | | 0.157 | | 0.020 | 1.70×10^-15^ |
| rs9276427 | C | T | 0.489 | | 0.038 | | 0.007 | 3.40×10^-08^ |
| rs2071536 | T | C | 0.116 | | 0.067 | | 0.011 | 5.30×10^-10^ |
| rs1042151 | A | G | 0.840 | | 0.055 | | 0.010 | 2.10×10^-08^ |
| rs9480610 | A | G | 0.228 | | 0.055 | | 0.008 | 2.20×10^-11^ |
| rs7749390 | A | G | 0.617 | | 0.062 | | 0.007 | 3.30×10^-18^ |
| rs11757201 | C | G | 0.219 | | 0.053 | | 0.008 | 2.20×10^-10^ |
| rs10267578 | C | T | 0.576 | | 0.039 | | 0.007 | 1.70×10^-08^ |
| rs17552787 | T | C | 0.320 | | 0.072 | | 0.007 | 2.10×10^-22^ |
| rs7804185 | T | C | 0.601 | | 0.048 | | 0.007 | 8.70×10^-12^ |
| rs112629741 | T | C | 0.960 | | 0.096 | | 0.018 | 4.30×10^-08^ |
| rs3757387 | C | T | 0.454 | | 0.045 | | 0.007 | 5.50×10^-11^ |
| rs62491812 | C | A | 0.798 | | 0.050 | | 0.009 | 5.40×10^-09^ |
| rs11989430 | T | A | 0.159 | | 0.082 | | 0.009 | 2.30×10^-18^ |
| rs4466418 | G | A | 0.438 | | 0.050 | | 0.007 | 4.00×10^-13^ |
| rs1545536 | T | C | 0.220 | | 0.050 | | 0.008 | 1.30×10^-09^ |
| rs10817678 | G | A | 0.333 | | 0.053 | | 0.007 | 5.60×10^-13^ |
| rs1800973 | A | C | 0.061 | | 0.127 | | 0.014 | 7.90×10^-19^ |
| rs61955029 | G | T | 0.388 | | 0.045 | | 0.007 | 2.40×10^-10^ |
| rs2066844 | C | T | 0.953 | | 0.129 | | 0.016 | 1.70×10^-15^ |
| rs72798422 | T | C | 0.967 | | 0.108 | | 0.019 | 1.70×10^-08^ |
| rs896263 | G | C | 0.253 | | 0.054 | | 0.008 | 1.00×10^-11^ |
| rs56177354 | C | T | 0.941 | | 0.086 | | 0.015 | 8.40×10^-09^ |
| rs11649485 | A | G | 0.802 | | 0.081 | | 0.009 | 1.10×10^-20^ |
| rs7193275 | G | C | 0.157 | | 0.075 | | 0.010 | 7.20×10^-15^ |
| rs12232497 | C | T | 0.474 | | 0.041 | | 0.007 | 2.90×10^-09^ |
| rs10411970 | C | A | 0.520 | | 0.049 | | 0.007 | 1.00×10^-12^ |
| rs2305742 | A | C | 0.789 | | 0.060 | | 0.008 | 9.20×10^-13^ |
| rs144474740 | C | T | 0.941 | | 0.088 | | 0.015 | 3.40×10^-09^ |
| rs923621 | A | G | 0.424 | | 0.045 | | 0.007 | 7.20×10^-11^ |
| rs2902941 | A | G | 0.645 | | 0.043 | | 0.007 | 1.40×10^-09^ |
| rs913678 | C | T | 0.332 | | 0.061 | | 0.007 | 6.90×10^-17^ |

**Table S13. 64 valid instrumental variables used for Mendelian randomization analysis of mouth ulcers (Exposure) on autism spectrum disorder (Outcome).**

| **SNP** | **Effect allele** | **Non-effect allele** | | **Effect allele frequency** | | **Beta** | **SE** | **P** |
| --- | --- | --- | --- | --- | --- | --- | --- | --- |
| rs11204668 | G | A | 0.436 | | 0.044 | | 0.007 | 3.00×10^-10^ |
| rs3813961 | G | C | 0.015 | | 0.193 | | 0.029 | 3.60×10^-11^ |
| rs17015865 | G | A | 0.743 | | 0.048 | | 0.008 | 1.10×10^-09^ |
| rs4845140 | C | T | 0.957 | | 0.131 | | 0.017 | 2.20×10^-14^ |
| rs2232359 | G | A | 0.083 | | 0.101 | | 0.012 | 4.00×10^-16^ |
| rs35268627 | C | T | 0.124 | | 0.059 | | 0.010 | 1.80×10^-08^ |
| rs16823566 | G | T | 0.921 | | 0.087 | | 0.013 | 1.00×10^-11^ |
| rs11684030 | G | A | 0.368 | | 0.072 | | 0.007 | 3.00×10^-24^ |
| rs17712835 | T | C | 0.963 | | 0.112 | | 0.018 | 1.50×10^-09^ |
| rs1491958 | T | C | 0.080 | | 0.086 | | 0.013 | 1.00×10^-11^ |
| rs12629300 | T | A | 0.174 | | 0.085 | | 0.009 | 1.00×10^-20^ |
| rs73156502 | T | C | 0.023 | | 0.131 | | 0.023 | 1.50×10^-08^ |
| rs145743887 | T | A | 0.014 | | 0.202 | | 0.030 | 3.20×10^-11^ |
| rs150921465 | T | G | 0.016 | | 0.214 | | 0.028 | 4.30×10^-14^ |
| rs116295688 | T | G | 0.025 | | 0.162 | | 0.024 | 7.70×10^-12^ |
| rs7617394 | C | T | 0.639 | | 0.071 | | 0.007 | 1.90×10^-22^ |
| rs6805758 | A | C | 0.669 | | 0.065 | | 0.007 | 5.80×10^-19^ |
| rs7645203 | C | T | 0.595 | | 0.084 | | 0.007 | 4.20×10^-33^ |
| rs73170558 | C | T | 0.904 | | 0.087 | | 0.012 | 1.90×10^-13^ |
| rs668998 | A | G | 0.562 | | 0.071 | | 0.007 | 8.00×10^-25^ |
| rs4680564 | A | G | 0.609 | | 0.056 | | 0.007 | 1.90×10^-15^ |
| rs55667203 | T | C | 0.169 | | 0.097 | | 0.009 | 2.60×10^-25^ |
| rs145925847 | A | T | 0.012 | | 0.226 | | 0.032 | 2.10×10^-12^ |
| rs4699030 | G | C | 0.582 | | 0.041 | | 0.007 | 3.10×10^-09^ |
| rs62351974 | C | T | 0.711 | | 0.044 | | 0.008 | 7.80×10^-09^ |
| rs4235479 | C | T | 0.501 | | 0.049 | | 0.007 | 8.10×10^-13^ |
| rs77630070 | G | T | 0.817 | | 0.052 | | 0.009 | 5.00×10^-09^ |
| rs4313034 | T | C | 0.817 | | 0.051 | | 0.009 | 1.10×10^-08^ |
| rs2517664 | C | T | 0.813 | | 0.059 | | 0.009 | 1.90×10^-11^ |
| rs78479381 | T | A | 0.098 | | 0.071 | | 0.012 | 7.10×10^-10^ |
| rs2074478 | T | C | 0.809 | | 0.053 | | 0.009 | 1.40×10^-09^ |
| rs2523589 | T | G | 0.496 | | 0.073 | | 0.007 | 2.70×10^-26^ |
| rs76518703 | G | A | 0.052 | | 0.129 | | 0.016 | 1.50×10^-15^ |
| rs138753323 | C | T | 0.033 | | 0.157 | | 0.020 | 1.70×10^-15^ |
| rs9276427 | C | T | 0.489 | | 0.038 | | 0.007 | 3.40×10^-08^ |
| rs2071536 | T | C | 0.116 | | 0.067 | | 0.011 | 5.30×10^-10^ |
| rs1042151 | A | G | 0.840 | | 0.055 | | 0.010 | 2.10×10^-08^ |
| rs9480610 | A | G | 0.228 | | 0.055 | | 0.008 | 2.20×10^-11^ |
| rs7749390 | A | G | 0.617 | | 0.062 | | 0.007 | 3.30×10^-18^ |
| rs11757201 | C | G | 0.219 | | 0.053 | | 0.008 | 2.20×10^-10^ |
| rs10267578 | C | T | 0.576 | | 0.039 | | 0.007 | 1.70×10^-08^ |
| rs17552787 | T | C | 0.320 | | 0.072 | | 0.007 | 2.10×10^-22^ |
| rs7804185 | T | C | 0.601 | | 0.048 | | 0.007 | 8.70×10^-12^ |
| rs112629741 | T | C | 0.960 | | 0.096 | | 0.018 | 4.30×10^-08^ |
| rs3757387 | C | T | 0.454 | | 0.045 | | 0.007 | 5.50×10^-11^ |
| rs62491812 | C | A | 0.798 | | 0.050 | | 0.009 | 5.40×10^-09^ |
| rs11989430 | T | A | 0.159 | | 0.082 | | 0.009 | 2.30×10^-18^ |
| rs4466418 | G | A | 0.438 | | 0.050 | | 0.007 | 4.00×10^-13^ |
| rs1545536 | T | C | 0.220 | | 0.050 | | 0.008 | 1.30×10^-09^ |
| rs10817678 | G | A | 0.333 | | 0.053 | | 0.007 | 5.60×10^-13^ |
| rs61955029 | G | T | 0.388 | | 0.045 | | 0.007 | 2.40×10^-10^ |
| rs2066844 | C | T | 0.953 | | 0.129 | | 0.016 | 1.70×10^-15^ |
| rs72798422 | T | C | 0.967 | | 0.108 | | 0.019 | 1.70×10^-08^ |
| rs896263 | G | C | 0.253 | | 0.054 | | 0.008 | 1.00×10^-11^ |
| rs56177354 | C | T | 0.941 | | 0.086 | | 0.015 | 8.40×10^-09^ |
| rs11649485 | A | G | 0.802 | | 0.081 | | 0.009 | 1.10×10^-20^ |
| rs7193275 | G | C | 0.157 | | 0.075 | | 0.010 | 7.20×10^-15^ |
| rs12232497 | C | T | 0.474 | | 0.041 | | 0.007 | 2.90×10^-09^ |
| rs10411970 | C | A | 0.520 | | 0.049 | | 0.007 | 1.00×10^-12^ |
| rs2305742 | A | C | 0.789 | | 0.060 | | 0.008 | 9.20×10^-13^ |
| rs144474740 | C | T | 0.941 | | 0.088 | | 0.015 | 3.40×10^-09^ |
| rs923621 | A | G | 0.424 | | 0.045 | | 0.007 | 7.20×10^-11^ |
| rs2902941 | A | G | 0.645 | | 0.043 | | 0.007 | 1.40×10^-09^ |
| rs913678 | C | T | 0.332 | | 0.061 | | 0.007 | 6.90×10^-17^ |

**Table S14. 59 valid instrumental variables used for Mendelian randomization analysis of mouth ulcers (Exposure) on bipolar disorder (Outcome).**

| **SNP** | **Effect allele** | **Non-effect allele** | | **Effect allele frequency** | | **Beta** | **SE** | ***P*** |
| --- | --- | --- | --- | --- | --- | --- | --- | --- |
| rs11204668 | G | A | 0.436 | | 0.044 | | 0.007 | 3.00×10^-10^ |
| rs2759663 | C | G | 0.779 | | 0.045 | | 0.008 | 3.80×10^-08^ |
| rs3813961 | G | C | 0.015 | | 0.193 | | 0.029 | 3.60×10^-11^ |
| rs17015865 | G | A | 0.743 | | 0.048 | | 0.008 | 1.10×10^-09^ |
| rs4845140 | C | T | 0.957 | | 0.131 | | 0.017 | 2.20×10^-14^ |
| rs2232359 | G | A | 0.083 | | 0.101 | | 0.012 | 4.00×10^-16^ |
| rs35268627 | C | T | 0.124 | | 0.059 | | 0.010 | 1.80×10^-08^ |
| rs16823566 | G | T | 0.921 | | 0.087 | | 0.013 | 1.00×10^-11^ |
| rs11684030 | G | A | 0.368 | | 0.072 | | 0.007 | 3.00×10^-24^ |
| rs17712835 | T | C | 0.963 | | 0.112 | | 0.018 | 1.50×10^-09^ |
| rs1491958 | T | C | 0.080 | | 0.086 | | 0.013 | 1.00×10^-11^ |
| rs12629300 | T | A | 0.174 | | 0.085 | | 0.009 | 1.00×10^-20^ |
| rs73156502 | T | C | 0.023 | | 0.131 | | 0.023 | 1.50×10^-08^ |
| rs145743887 | T | A | 0.014 | | 0.202 | | 0.030 | 3.20×10^-11^ |
| rs150921465 | T | G | 0.016 | | 0.214 | | 0.028 | 4.30×10^-14^ |
| rs116295688 | T | G | 0.025 | | 0.162 | | 0.024 | 7.70×10^-12^ |
| rs11710258 | G | A | 0.652 | | 0.041 | | 0.007 | 1.40×10^-08^ |
| rs7617394 | C | T | 0.639 | | 0.071 | | 0.007 | 1.90×10^-22^ |
| rs73170558 | C | T | 0.904 | | 0.087 | | 0.012 | 1.90×10^-13^ |
| rs668998 | A | G | 0.562 | | 0.071 | | 0.007 | 8.00×10^-25^ |
| rs4680564 | A | G | 0.609 | | 0.056 | | 0.007 | 1.90×10^-15^ |
| rs55667203 | T | C | 0.169 | | 0.097 | | 0.009 | 2.60×10^-25^ |
| rs150383292 | T | C | 0.016 | | 0.212 | | 0.028 | 5.30×10^-14^ |
| rs4699030 | G | C | 0.582 | | 0.041 | | 0.007 | 3.10×10^-09^ |
| rs62351974 | C | T | 0.711 | | 0.044 | | 0.008 | 7.80×10^-09^ |
| rs4235479 | C | T | 0.501 | | 0.049 | | 0.007 | 8.10×10^-13^ |
| rs4921484 | C | T | 0.679 | | 0.047 | | 0.007 | 2.00×10^-10^ |
| rs77630070 | G | T | 0.817 | | 0.052 | | 0.009 | 5.00×10^-09^ |
| rs78479381 | T | A | 0.098 | | 0.071 | | 0.012 | 7.10×10^-10^ |
| rs2074478 | T | C | 0.809 | | 0.053 | | 0.009 | 1.40×10^-09^ |
| rs2523589 | T | G | 0.496 | | 0.073 | | 0.007 | 2.70×10^-26^ |
| rs138753323 | C | T | 0.033 | | 0.157 | | 0.020 | 1.70×10^-15^ |
| rs2071536 | T | C | 0.116 | | 0.067 | | 0.011 | 5.30×10^-10^ |
| rs9480610 | A | G | 0.228 | | 0.055 | | 0.008 | 2.20×10^-11^ |
| rs7749390 | A | G | 0.617 | | 0.062 | | 0.007 | 3.30×10^-18^ |
| rs11757201 | C | G | 0.219 | | 0.053 | | 0.008 | 2.20×10^-10^ |
| rs10267578 | C | T | 0.576 | | 0.039 | | 0.007 | 1.70×10^-08^ |
| rs17552787 | T | C | 0.320 | | 0.072 | | 0.007 | 2.10×10^-22^ |
| rs7804185 | T | C | 0.601 | | 0.048 | | 0.007 | 8.70×10^-12^ |
| rs3757387 | C | T | 0.454 | | 0.045 | | 0.007 | 5.50×10^-11^ |
| rs62491812 | C | A | 0.798 | | 0.050 | | 0.009 | 5.40×10^-09^ |
| rs11989430 | T | A | 0.159 | | 0.082 | | 0.009 | 2.30×10^-18^ |
| rs4466418 | G | A | 0.438 | | 0.050 | | 0.007 | 4.00×10^-13^ |
| rs1545536 | T | C | 0.220 | | 0.050 | | 0.008 | 1.30×10^-09^ |
| rs10817678 | G | A | 0.333 | | 0.053 | | 0.007 | 5.60×10^-13^ |
| rs1800973 | A | C | 0.061 | | 0.127 | | 0.014 | 7.90×10^-19^ |
| rs61955029 | G | T | 0.388 | | 0.045 | | 0.007 | 2.40×10^-10^ |
| rs2066844 | C | T | 0.953 | | 0.129 | | 0.016 | 1.70×10^-15^ |
| rs72798422 | T | C | 0.967 | | 0.108 | | 0.019 | 1.70×10^-08^ |
| rs896263 | G | C | 0.253 | | 0.054 | | 0.008 | 1.00×10^-11^ |
| rs56177354 | C | T | 0.941 | | 0.086 | | 0.015 | 8.40×10^-09^ |
| rs11649485 | A | G | 0.802 | | 0.081 | | 0.009 | 1.10×10^-20^ |
| rs7193275 | G | C | 0.157 | | 0.075 | | 0.010 | 7.20×10^-15^ |
| rs10411970 | C | A | 0.520 | | 0.049 | | 0.007 | 1.00×10^-12^ |
| rs2305742 | A | C | 0.789 | | 0.060 | | 0.008 | 9.20×10^-13^ |
| rs144474740 | C | T | 0.941 | | 0.088 | | 0.015 | 3.40×10^-09^ |
| rs923621 | A | G | 0.424 | | 0.045 | | 0.007 | 7.20×10^-11^ |
| rs2902941 | A | G | 0.645 | | 0.043 | | 0.007 | 1.40×10^-09^ |
| rs913678 | C | T | 0.332 | | 0.061 | | 0.007 | 6.90×10^-17^ |

**Table S15. 61 valid instrumental variables used for Mendelian randomization analysis of mouth ulcers (Exposure) on insomnia (Outcome).**

| **SNP** | **Effect allele** | **Non-effect allele** | **Effect allele frequency** | | **Beta** | **SE** | ***P*** |
| --- | --- | --- | --- | --- | --- | --- | --- |
| rs11204668 | G | A | 0.436 | 0.044 | | 0.007 | 3.00×10^-10^ |
| rs2759663 | C | G | 0.779 | 0.045 | | 0.008 | 3.80×10^-08^ |
| rs17015865 | G | A | 0.743 | 0.048 | | 0.008 | 1.10×10^-09^ |
| rs4845140 | C | T | 0.957 | 0.131 | | 0.017 | 2.20×10^-14^ |
| rs2232359 | G | A | 0.083 | 0.101 | | 0.012 | 4.00×10^-16^ |
| rs35268627 | C | T | 0.124 | 0.059 | | 0.010 | 1.80×10^-08^ |
| rs16823566 | G | T | 0.921 | 0.087 | | 0.013 | 1.00×10^-11^ |
| rs11684030 | G | A | 0.368 | 0.072 | | 0.007 | 3.00×10^-24^ |
| rs17712835 | T | C | 0.963 | 0.112 | | 0.018 | 1.50×10^-09^ |
| rs1491958 | T | C | 0.080 | 0.086 | | 0.013 | 1.00×10^-11^ |
| rs12629300 | T | A | 0.174 | 0.085 | | 0.009 | 1.00×10^-20^ |
| rs73156502 | T | C | 0.023 | 0.131 | | 0.023 | 1.50×10^-08^ |
| rs145743887 | T | A | 0.014 | 0.202 | | 0.030 | 3.20×10^-11^ |
| rs150921465 | T | G | 0.016 | 0.214 | | 0.028 | 4.30×10^-14^ |
| rs11710258 | G | A | 0.652 | 0.041 | | 0.007 | 1.40×10^-08^ |
| rs6805758 | A | C | 0.669 | 0.065 | | 0.007 | 5.80×10^-19^ |
| rs7645203 | C | T | 0.595 | 0.084 | | 0.007 | 4.20×10^-33^ |
| rs73170558 | C | T | 0.904 | 0.087 | | 0.012 | 1.90×10^-13^ |
| rs668998 | A | G | 0.562 | 0.071 | | 0.007 | 8.00×10^-25^ |
| rs55667203 | T | C | 0.169 | 0.097 | | 0.009 | 2.60×10^-25^ |
| rs145925847 | A | T | 0.012 | 0.226 | | 0.032 | 2.10×10^-12^ |
| rs150383292 | T | C | 0.016 | 0.212 | | 0.028 | 5.30×10^-14^ |
| rs4699030 | G | C | 0.582 | 0.041 | | 0.007 | 3.10×10^-09^ |
| rs62351974 | C | T | 0.711 | 0.044 | | 0.008 | 7.80×10^-09^ |
| rs4235479 | C | T | 0.501 | 0.049 | | 0.007 | 8.10×10^-13^ |
| rs4921484 | C | T | 0.679 | 0.047 | | 0.007 | 2.00×10^-10^ |
| rs77630070 | G | T | 0.817 | 0.052 | | 0.009 | 5.00×10^-09^ |
| rs4313034 | T | C | 0.817 | 0.051 | | 0.009 | 1.10×10^-08^ |
| rs78479381 | T | A | 0.098 | 0.071 | | 0.012 | 7.10×10^-10^ |
| rs2523589 | T | G | 0.496 | 0.073 | | 0.007 | 2.70×10^-26^ |
| rs76518703 | G | A | 0.052 | 0.129 | | 0.016 | 1.50×10^-15^ |
| rs138753323 | C | T | 0.033 | 0.157 | | 0.020 | 1.70×10^-15^ |
| rs9276427 | C | T | 0.489 | 0.038 | | 0.007 | 3.40×10^-08^ |
| rs2071536 | T | C | 0.116 | 0.067 | | 0.011 | 5.30×10^-10^ |
| rs9480610 | A | G | 0.228 | 0.055 | | 0.008 | 2.20×10^-11^ |
| rs7749390 | A | G | 0.617 | 0.062 | | 0.007 | 3.30×10^-18^ |
| rs11757201 | C | G | 0.219 | 0.053 | | 0.008 | 2.20×10^-10^ |
| rs10267578 | C | T | 0.576 | 0.039 | | 0.007 | 1.70×10^-08^ |
| rs17552787 | T | C | 0.320 | 0.072 | | 0.007 | 2.10×10^-22^ |
| rs7804185 | T | C | 0.601 | 0.048 | | 0.007 | 8.70×10^-12^ |
| rs112629741 | T | C | 0.960 | 0.096 | | 0.018 | 4.30×10^-08^ |
| rs3757387 | C | T | 0.454 | 0.045 | | 0.007 | 5.50×10^-11^ |
| rs62491812 | C | A | 0.798 | 0.050 | | 0.009 | 5.40×10^-09^ |
| rs11989430 | T | A | 0.159 | 0.082 | | 0.009 | 2.30×10^-18^ |
| rs4466418 | G | A | 0.438 | 0.050 | | 0.007 | 4.00×10^-13^ |
| rs1545536 | T | C | 0.220 | 0.050 | | 0.008 | 1.30×10^-09^ |
| rs10817678 | G | A | 0.333 | 0.053 | | 0.007 | 5.60×10^-13^ |
| rs1800973 | A | C | 0.061 | 0.127 | | 0.014 | 7.90×10^-19^ |
| rs61955029 | G | T | 0.388 | 0.045 | | 0.007 | 2.40×10^-10^ |
| rs72798422 | T | C | 0.967 | 0.108 | | 0.019 | 1.70×10^-08^ |
| rs896263 | G | C | 0.253 | 0.054 | | 0.008 | 1.00×10^-11^ |
| rs56177354 | C | T | 0.941 | 0.086 | | 0.015 | 8.40×10^-09^ |
| rs11649485 | A | G | 0.802 | 0.081 | | 0.009 | 1.10×10^-20^ |
| rs12232497 | C | T | 0.474 | 0.041 | | 0.007 | 2.90×10^-09^ |
| rs79742625 | T | C | 0.205 | 0.061 | | 0.009 | 1.90×10^-12^ |
| rs10411970 | C | A | 0.520 | 0.049 | | 0.007 | 1.00×10^-12^ |
| rs2305742 | A | C | 0.789 | 0.060 | | 0.008 | 9.20×10^-13^ |
| rs144474740 | C | T | 0.941 | 0.088 | | 0.015 | 3.40×10^-09^ |
| rs923621 | A | G | 0.424 | 0.045 | | 0.007 | 7.20×10^-11^ |
| rs2902941 | A | G | 0.645 | 0.043 | | 0.007 | 1.40×10^-09^ |
| rs913678 | C | T | 0.332 | 0.061 | | 0.007 | 6.90×10^-17^ |

**Table S16. 54 valid instrumental variables used for Mendelian randomization analysis of mouth ulcers (Exposure) on major depressive disorder (Outcome).**

| **SNP** | **Effect allele** | **Non-effect allele** | | **Effect allele frequency** | | **Beta** | **SE** | ***P*** |
| --- | --- | --- | --- | --- | --- | --- | --- | --- |
| rs11204668 | G | A | 0.436 | | 0.044 | | 0.007 | 3.00×10^-10^ |
| rs3813961 | G | C | 0.015 | | 0.193 | | 0.029 | 3.60×10^-11^ |
| rs17015865 | G | A | 0.743 | | 0.048 | | 0.008 | 1.10×10^-09^ |
| rs4845140 | C | T | 0.957 | | 0.131 | | 0.017 | 2.20×10^-14^ |
| rs2232359 | G | A | 0.083 | | 0.101 | | 0.012 | 4.00×10^-16^ |
| rs35268627 | C | T | 0.124 | | 0.059 | | 0.010 | 1.80×10^-08^ |
| rs16823566 | G | T | 0.921 | | 0.087 | | 0.013 | 1.00×10^-11^ |
| rs17712835 | T | C | 0.963 | | 0.112 | | 0.018 | 1.50×10^-09^ |
| rs1491958 | T | C | 0.080 | | 0.086 | | 0.013 | 1.00×10^-11^ |
| rs12629300 | T | A | 0.174 | | 0.085 | | 0.009 | 1.00×10^-20^ |
| rs73156502 | T | C | 0.023 | | 0.131 | | 0.023 | 1.50×10^-08^ |
| rs145743887 | T | A | 0.014 | | 0.202 | | 0.030 | 3.20×10^-11^ |
| rs150921465 | T | G | 0.016 | | 0.214 | | 0.028 | 4.30×10^-14^ |
| rs116295688 | T | G | 0.025 | | 0.162 | | 0.024 | 7.70×10^-12^ |
| rs11710258 | G | A | 0.652 | | 0.041 | | 0.007 | 1.40×10^-08^ |
| rs7617394 | C | T | 0.639 | | 0.071 | | 0.007 | 1.90×10^-22^ |
| rs6805758 | A | C | 0.669 | | 0.065 | | 0.007 | 5.80×10^-19^ |
| rs73170558 | C | T | 0.904 | | 0.087 | | 0.012 | 1.90×10^-13^ |
| rs668998 | A | G | 0.562 | | 0.071 | | 0.007 | 8.00×10^-25^ |
| rs4680564 | A | G | 0.609 | | 0.056 | | 0.007 | 1.90×10^-15^ |
| rs55667203 | T | C | 0.169 | | 0.097 | | 0.009 | 2.60×10^-25^ |
| rs145925847 | A | T | 0.012 | | 0.226 | | 0.032 | 2.10×10^-12^ |
| rs150383292 | T | C | 0.016 | | 0.212 | | 0.028 | 5.30×10^-14^ |
| rs4699030 | G | C | 0.582 | | 0.041 | | 0.007 | 3.10×10^-09^ |
| rs62351974 | C | T | 0.711 | | 0.044 | | 0.008 | 7.80×10^-09^ |
| rs4235479 | C | T | 0.501 | | 0.049 | | 0.007 | 8.10×10^-13^ |
| rs4921484 | C | T | 0.679 | | 0.047 | | 0.007 | 2.00×10^-10^ |
| rs2074478 | T | C | 0.809 | | 0.053 | | 0.009 | 1.40×10^-09^ |
| rs138753323 | C | T | 0.033 | | 0.157 | | 0.020 | 1.70×10^-15^ |
| rs2071536 | T | C | 0.116 | | 0.067 | | 0.011 | 5.30×10^-10^ |
| rs9480610 | A | G | 0.228 | | 0.055 | | 0.008 | 2.20×10^-11^ |
| rs7749390 | A | G | 0.617 | | 0.062 | | 0.007 | 3.30×10^-18^ |
| rs11757201 | C | G | 0.219 | | 0.053 | | 0.008 | 2.20×10^-10^ |
| rs10267578 | C | T | 0.576 | | 0.039 | | 0.007 | 1.70×10^-08^ |
| rs17552787 | T | C | 0.320 | | 0.072 | | 0.007 | 2.10×10^-22^ |
| rs112629741 | T | C | 0.960 | | 0.096 | | 0.018 | 4.30×10^-08^ |
| rs3757387 | C | T | 0.454 | | 0.045 | | 0.007 | 5.50×10^-11^ |
| rs62491812 | C | A | 0.798 | | 0.050 | | 0.009 | 5.40×10^-09^ |
| rs11989430 | T | A | 0.159 | | 0.082 | | 0.009 | 2.30×10^-18^ |
| rs4466418 | G | A | 0.438 | | 0.050 | | 0.007 | 4.00×10^-13^ |
| rs1545536 | T | C | 0.220 | | 0.050 | | 0.008 | 1.30×10^-09^ |
| rs10817678 | G | A | 0.333 | | 0.053 | | 0.007 | 5.60×10^-13^ |
| rs61955029 | G | T | 0.388 | | 0.045 | | 0.007 | 2.40×10^-10^ |
| rs2066844 | C | T | 0.953 | | 0.129 | | 0.016 | 1.70×10^-15^ |
| rs72798422 | T | C | 0.967 | | 0.108 | | 0.019 | 1.70×10^-08^ |
| rs896263 | G | C | 0.253 | | 0.054 | | 0.008 | 1.00×10^-11^ |
| rs56177354 | C | T | 0.941 | | 0.086 | | 0.015 | 8.40×10^-09^ |
| rs7193275 | G | C | 0.157 | | 0.075 | | 0.010 | 7.20×10^-15^ |
| rs12232497 | C | T | 0.474 | | 0.041 | | 0.007 | 2.90×10^-09^ |
| rs10411970 | C | A | 0.520 | | 0.049 | | 0.007 | 1.00×10^-12^ |
| rs2305742 | A | C | 0.789 | | 0.060 | | 0.008 | 9.20×10^-13^ |
| rs923621 | A | G | 0.424 | | 0.045 | | 0.007 | 7.20×10^-11^ |
| rs2902941 | A | G | 0.645 | | 0.043 | | 0.007 | 1.40×10^-09^ |
| rs913678 | C | T | 0.332 | | 0.061 | | 0.007 | 6.90×10^-17^ |

**Table S17. 54 valid instrumental variables used for Mendelian randomization analysis of mouth ulcers (Exposure) on neuroticism (Outcome).**

| **SNP** | **Effect allele** | **Non-effect allele** | **Effect allele frequency** | **Beta** | **SE** | ***P*** |
| --- | --- | --- | --- | --- | --- | --- |
| rs11204668 | G | A | 0.436 | 0.044 | 0.007 | 3.00×10^-10^ |
| rs17015865 | G | A | 0.743 | 0.048 | 0.008 | 1.10×10^-09^ |
| rs4845140 | C | T | 0.957 | 0.131 | 0.017 | 2.20×10^-14^ |
| rs2232359 | G | A | 0.083 | 0.101 | 0.012 | 4.00×10^-16^ |
| rs35268627 | C | T | 0.124 | 0.059 | 0.010 | 1.80×10^-08^ |
| rs16823566 | G | T | 0.921 | 0.087 | 0.013 | 1.00×10^-11^ |
| rs11684030 | G | A | 0.368 | 0.072 | 0.007 | 3.00×10^-24^ |
| rs17712835 | T | C | 0.963 | 0.112 | 0.018 | 1.50×10^-09^ |
| rs1491958 | T | C | 0.080 | 0.086 | 0.013 | 1.00×10^-11^ |
| rs12629300 | T | A | 0.174 | 0.085 | 0.009 | 1.00×10^-20^ |
| rs11710258 | G | A | 0.652 | 0.041 | 0.007 | 1.40×10^-08^ |
| rs7617394 | C | T | 0.639 | 0.071 | 0.007 | 1.90×10^-22^ |
| rs6805758 | A | C | 0.669 | 0.065 | 0.007 | 5.80×10^-19^ |
| rs7645203 | C | T | 0.595 | 0.084 | 0.007 | 4.20×10^-33^ |
| rs73170558 | C | T | 0.904 | 0.087 | 0.012 | 1.90×10^-13^ |
| rs668998 | A | G | 0.562 | 0.071 | 0.007 | 8.00×10^-25^ |
| rs4680564 | A | G | 0.609 | 0.056 | 0.007 | 1.90×10^-15^ |
| rs55667203 | T | C | 0.169 | 0.097 | 0.009 | 2.60×10^-25^ |
| rs4699030 | G | C | 0.582 | 0.041 | 0.007 | 3.10×10^-09^ |
| rs62351974 | C | T | 0.711 | 0.044 | 0.008 | 7.80×10^-09^ |
| rs4235479 | C | T | 0.501 | 0.049 | 0.007 | 8.10×10^-13^ |
| rs4921484 | C | T | 0.679 | 0.047 | 0.007 | 2.00×10^-10^ |
| rs77630070 | G | T | 0.817 | 0.052 | 0.009 | 5.00×10^-09^ |
| rs78479381 | T | A | 0.098 | 0.071 | 0.012 | 7.10×10^-10^ |
| rs2074478 | T | C | 0.809 | 0.053 | 0.009 | 1.40×10^-09^ |
| rs2523589 | T | G | 0.496 | 0.073 | 0.007 | 2.70×10^-26^ |
| rs2071536 | T | C | 0.116 | 0.067 | 0.011 | 5.30×10^-10^ |
| rs9480610 | A | G | 0.228 | 0.055 | 0.008 | 2.20×10^-11^ |
| rs7749390 | A | G | 0.617 | 0.062 | 0.007 | 3.30×10^-18^ |
| rs11757201 | C | G | 0.219 | 0.053 | 0.008 | 2.20×10^-10^ |
| rs10267578 | C | T | 0.576 | 0.039 | 0.007 | 1.70×10^-08^ |
| rs17552787 | T | C | 0.320 | 0.072 | 0.007 | 2.10×10^-22^ |
| rs7804185 | T | C | 0.601 | 0.048 | 0.007 | 8.70×10^-12^ |
| rs112629741 | T | C | 0.960 | 0.096 | 0.018 | 4.30×10^-08^ |
| rs3757387 | C | T | 0.454 | 0.045 | 0.007 | 5.50×10^-11^ |
| rs62491812 | C | A | 0.798 | 0.050 | 0.009 | 5.40×10^-09^ |
| rs11989430 | T | A | 0.159 | 0.082 | 0.009 | 2.30×10^-18^ |
| rs4466418 | G | A | 0.438 | 0.050 | 0.007 | 4.00×10^-13^ |
| rs1545536 | T | C | 0.220 | 0.050 | 0.008 | 1.30×10^-09^ |
| rs10817678 | G | A | 0.333 | 0.053 | 0.007 | 5.60×10^-13^ |
| rs1800973 | A | C | 0.061 | 0.127 | 0.014 | 7.90×10^-19^ |
| rs61955029 | G | T | 0.388 | 0.045 | 0.007 | 2.40×10^-10^ |
| rs2066844 | C | T | 0.953 | 0.129 | 0.016 | 1.70×10^-15^ |
| rs72798422 | T | C | 0.967 | 0.108 | 0.019 | 1.70×10^-08^ |
| rs896263 | G | C | 0.253 | 0.054 | 0.008 | 1.00×10^-11^ |
| rs56177354 | C | T | 0.941 | 0.086 | 0.015 | 8.40×10^-09^ |
| rs11649485 | A | G | 0.802 | 0.081 | 0.009 | 1.10×10^-20^ |
| rs7193275 | G | C | 0.157 | 0.075 | 0.010 | 7.20×10^-15^ |
| rs12232497 | C | T | 0.474 | 0.041 | 0.007 | 2.90×10^-09^ |
| rs10411970 | C | A | 0.520 | 0.049 | 0.007 | 1.00×10^-12^ |
| rs2305742 | A | C | 0.789 | 0.060 | 0.008 | 9.20×10^-13^ |
| rs144474740 | C | T | 0.941 | 0.088 | 0.015 | 3.40×10^-09^ |
| rs923621 | A | G | 0.424 | 0.045 | 0.007 | 7.20×10^-11^ |
| rs2902941 | A | G | 0.645 | 0.043 | 0.007 | 1.40×10^-09^ |

**Table S18. 60 valid instrumental variables used for Mendelian randomization analysis of mouth ulcers (Exposure) on schizophrenia (Outcome).**

| **SNP** | **Effect allele** | **Non-effect allele** | | **Effect allele frequency** | | **Beta** | **SE** | ***P*** |
| --- | --- | --- | --- | --- | --- | --- | --- | --- |
| rs11204668 | G | A | 0.436 | | 0.044 | | 0.007 | 3.00×10^-10^ |
| rs3813961 | G | C | 0.015 | | 0.193 | | 0.029 | 3.60×10^-11^ |
| rs17015865 | G | A | 0.743 | | 0.048 | | 0.008 | 1.10×10^-09^ |
| rs4845140 | C | T | 0.957 | | 0.131 | | 0.017 | 2.20×10^-14^ |
| rs2232359 | G | A | 0.083 | | 0.101 | | 0.012 | 4.00×10^-16^ |
| rs35268627 | C | T | 0.124 | | 0.059 | | 0.010 | 1.80×10^-08^ |
| rs16823566 | G | T | 0.921 | | 0.087 | | 0.013 | 1.00×10^-11^ |
| rs11684030 | G | A | 0.368 | | 0.072 | | 0.007 | 3.00×10^-24^ |
| rs17712835 | T | C | 0.963 | | 0.112 | | 0.018 | 1.50×10^-09^ |
| rs1491958 | T | C | 0.080 | | 0.086 | | 0.013 | 1.00×10^-11^ |
| rs12629300 | T | A | 0.174 | | 0.085 | | 0.009 | 1.00×10^-20^ |
| rs73156502 | T | C | 0.023 | | 0.131 | | 0.023 | 1.50×10^-08^ |
| rs145743887 | T | A | 0.014 | | 0.202 | | 0.030 | 3.20×10^-11^ |
| rs150921465 | T | G | 0.016 | | 0.214 | | 0.028 | 4.30×10^-14^ |
| rs116295688 | T | G | 0.025 | | 0.162 | | 0.024 | 7.70×10^-12^ |
| rs11710258 | G | A | 0.652 | | 0.041 | | 0.007 | 1.40×10^-08^ |
| rs7617394 | C | T | 0.639 | | 0.071 | | 0.007 | 1.90×10^-22^ |
| rs6805758 | A | C | 0.669 | | 0.065 | | 0.007 | 5.80×10^-19^ |
| rs7645203 | C | T | 0.595 | | 0.084 | | 0.007 | 4.20×10^-33^ |
| rs73170558 | C | T | 0.904 | | 0.087 | | 0.012 | 1.90×10^-13^ |
| rs668998 | A | G | 0.562 | | 0.071 | | 0.007 | 8.00×10^-25^ |
| rs4680564 | A | G | 0.609 | | 0.056 | | 0.007 | 1.90×10^-15^ |
| rs55667203 | T | C | 0.169 | | 0.097 | | 0.009 | 2.60×10^-25^ |
| rs145925847 | A | T | 0.012 | | 0.226 | | 0.032 | 2.10×10^-12^ |
| rs150383292 | T | C | 0.016 | | 0.212 | | 0.028 | 5.30×10^-14^ |
| rs4699030 | G | C | 0.582 | | 0.041 | | 0.007 | 3.10×10^-09^ |
| rs62351974 | C | T | 0.711 | | 0.044 | | 0.008 | 7.80×10^-09^ |
| rs4235479 | C | T | 0.501 | | 0.049 | | 0.007 | 8.10×10^-13^ |
| rs77630070 | G | T | 0.817 | | 0.052 | | 0.009 | 5.00×10^-09^ |
| rs78479381 | T | A | 0.098 | | 0.071 | | 0.012 | 7.10×10^-10^ |
| rs76518703 | G | A | 0.052 | | 0.129 | | 0.016 | 1.50×10^-15^ |
| rs138753323 | C | T | 0.033 | | 0.157 | | 0.020 | 1.70×10^-15^ |
| rs9276427 | C | T | 0.489 | | 0.038 | | 0.007 | 3.40×10^-08^ |
| rs2071536 | T | C | 0.116 | | 0.067 | | 0.011 | 5.30×10^-10^ |
| rs1042151 | A | G | 0.840 | | 0.055 | | 0.010 | 2.10×10^-08^ |
| rs7749390 | A | G | 0.617 | | 0.062 | | 0.007 | 3.30×10^-18^ |
| rs11757201 | C | G | 0.219 | | 0.053 | | 0.008 | 2.20×10^-10^ |
| rs10267578 | C | T | 0.576 | | 0.039 | | 0.007 | 1.70×10^-08^ |
| rs17552787 | T | C | 0.320 | | 0.072 | | 0.007 | 2.10×10^-22^ |
| rs7804185 | T | C | 0.601 | | 0.048 | | 0.007 | 8.70×10^-12^ |
| rs112629741 | T | C | 0.960 | | 0.096 | | 0.018 | 4.30×10^-08^ |
| rs3757387 | C | T | 0.454 | | 0.045 | | 0.007 | 5.50×10^-11^ |
| rs62491812 | C | A | 0.798 | | 0.050 | | 0.009 | 5.40×10^-09^ |
| rs11989430 | T | A | 0.159 | | 0.082 | | 0.009 | 2.30×10^-18^ |
| rs4466418 | G | A | 0.438 | | 0.050 | | 0.007 | 4.00×10^-13^ |
| rs1545536 | T | C | 0.220 | | 0.050 | | 0.008 | 1.30×10^-09^ |
| rs10817678 | G | A | 0.333 | | 0.053 | | 0.007 | 5.60×10^-13^ |
| rs1800973 | A | C | 0.061 | | 0.127 | | 0.014 | 7.90×10^-19^ |
| rs61955029 | G | T | 0.388 | | 0.045 | | 0.007 | 2.40×10^-10^ |
| rs2066844 | C | T | 0.953 | | 0.129 | | 0.016 | 1.70×10^-15^ |
| rs72798422 | T | C | 0.967 | | 0.108 | | 0.019 | 1.70×10^-08^ |
| rs11649485 | A | G | 0.802 | | 0.081 | | 0.009 | 1.10×10^-20^ |
| rs7193275 | G | C | 0.157 | | 0.075 | | 0.010 | 7.20×10^-15^ |
| rs12232497 | C | T | 0.474 | | 0.041 | | 0.007 | 2.90×10^-09^ |
| rs10411970 | C | A | 0.520 | | 0.049 | | 0.007 | 1.00×10^-12^ |
| rs2305742 | A | C | 0.789 | | 0.060 | | 0.008 | 9.20×10^-13^ |
| rs144474740 | C | T | 0.941 | | 0.088 | | 0.015 | 3.40×10^-09^ |
| rs923621 | A | G | 0.424 | | 0.045 | | 0.007 | 7.20×10^-11^ |
| rs2902941 | A | G | 0.645 | | 0.043 | | 0.007 | 1.40×10^-09^ |
| rs913678 | C | T | 0.332 | | 0.061 | | 0.007 | 6.90×10^-17^ |

**Table S19. 28 valid instrumental variables used for Mendelian randomization analysis of mouth ulcers (Exposure) on subjective wellbeing (Outcome).**

| **SNP** | **Effect allele** | **Non-effect allele** | | **Effect allele frequency** | | **Beta** | **SE** | ***P*** |
| --- | --- | --- | --- | --- | --- | --- | --- | --- |
| rs11204668 | G | A | 0.436 | | 0.044 | | 0.007 | 3.00×10^-10^ |
| rs2759663 | C | G | 0.779 | | 0.045 | | 0.008 | 3.80×10^-08^ |
| rs17015865 | G | A | 0.743 | | 0.048 | | 0.008 | 1.10×10^-09^ |
| rs4845140 | C | T | 0.957 | | 0.131 | | 0.017 | 2.20×10^-14^ |
| rs16823566 | G | T | 0.921 | | 0.087 | | 0.013 | 1.00×10^-11^ |
| rs1491958 | T | C | 0.080 | | 0.086 | | 0.013 | 1.00×10^-11^ |
| rs12629300 | T | A | 0.174 | | 0.085 | | 0.009 | 1.00×10^-20^ |
| rs11710258 | G | A | 0.652 | | 0.041 | | 0.007 | 1.40×10^-08^ |
| rs6805758 | A | C | 0.669 | | 0.065 | | 0.007 | 5.80×10^-19^ |
| rs668998 | A | G | 0.562 | | 0.071 | | 0.007 | 8.00×10^-25^ |
| rs4699030 | G | C | 0.582 | | 0.041 | | 0.007 | 3.10×10^-09^ |
| rs4235479 | C | T | 0.501 | | 0.049 | | 0.007 | 8.10×10^-13^ |
| rs4921484 | C | T | 0.679 | | 0.047 | | 0.007 | 2.00×10^-10^ |
| rs2074478 | T | C | 0.809 | | 0.053 | | 0.009 | 1.40×10^-09^ |
| rs2523589 | T | G | 0.496 | | 0.073 | | 0.007 | 2.70×10^-26^ |
| rs9480610 | A | G | 0.228 | | 0.055 | | 0.008 | 2.20×10^-11^ |
| rs7749390 | A | G | 0.617 | | 0.062 | | 0.007 | 3.30×10^-18^ |
| rs10267578 | C | T | 0.576 | | 0.039 | | 0.007 | 1.70×10^-08^ |
| rs4466418 | G | A | 0.438 | | 0.050 | | 0.007 | 4.00×10^-13^ |
| rs1545536 | T | C | 0.220 | | 0.050 | | 0.008 | 1.30×10^-09^ |
| rs10817678 | G | A | 0.333 | | 0.053 | | 0.007 | 5.60×10^-13^ |
| rs2066844 | C | T | 0.953 | | 0.129 | | 0.016 | 1.70×10^-15^ |
| rs896263 | G | C | 0.253 | | 0.054 | | 0.008 | 1.00×10^-11^ |
| rs7193275 | G | C | 0.157 | | 0.075 | | 0.010 | 7.20×10^-15^ |
| rs12232497 | C | T | 0.474 | | 0.041 | | 0.007 | 2.90×10^-09^ |
| rs2305742 | A | C | 0.789 | | 0.060 | | 0.008 | 9.20×10^-13^ |
| rs2902941 | A | G | 0.645 | | 0.043 | | 0.007 | 1.40×10^-09^ |
| rs913678 | C | T | 0.332 | | 0.061 | | 0.007 | 6.90×10^-17^ |

**Table S20. Power calculation for two-sample MR analyses of 10 psychiatric traits on mouth ulcers.**

| **Exposure** | **Outcome** | **Variance Explained by the instruments (*R*^2^)** | ***OR* (power = 80%)** |
| --- | --- | --- | --- |
| anxiety disorders | mouth ulcers | 0.2% | 1.742 |
| ADHD |  | 0.7% | 1.235 |
| ASD |  | 0.7% | 1.223 |
| BIP |  | 1.3% | 1.167 |
| insomnia |  | 2.6% | 1.116 |
| MDD |  | 0.3% | 1.349 |
| mood instability |  | 0.3% | 1.349 |
| neuroticism |  | 0.8% | 1.210 |
| SCZ |  | 4.3% | 1.088 |
| subject wellbeing |  | 0.4% | 0.715 |

ADHD, attention deficit/hyperactivity disorder; ASD, autism spectrum disorder; BIP, bipolar disorder; MDD, major depressive disorder; SCZ, schizophrenia; Power calculations were conducted using Burgess’ online calculator; *OR*, the required *OR* (in the unit of per standard deviation increment in exposure) to achieve 80% statistical power given a significance level of *α*=2.8×10^-3^ and the corresponding summary statistics.

**Table S21. Power calculation for two-sample MR analyses of mouth ulcers on 8 psychiatric traits.**

| **Exposure** | **Outcome** | **Variance Explained by the Instruments (*R*^2^)** | ***OR* (power = 80%)** |
| --- | --- | --- | --- |
| mouth ulcers | ADHD | 2.1% | 1.260 |
|  | ASD | 2.1% | 1.280 |
|  | BIP | 1.9% | 1.275 |
|  | insomnia | 1.9% | 1.198 |
|  | MDD | 1.6% | 1.157 |
|  | neuroticism | 1.8% | 1.071 |
|  | SCZ | 1.9% | 1.186 |
|  | subject wellbeing | 0.9% | 0.915 |

ADHD, attention deficit/hyperactivity disorder; ASD, autism spectrum disorder; BIP, bipolar disorder; MDD, major depressive disorder; SCZ, schizophrenia; Power calculations were conducted using Burgess’ online calculator; *OR*, the required *OR* (in the unit of per standard deviation increment in exposure) to achieve 80% statistical power given a significance level of *α*=2.8×10^-3^ and the corresponding summary statistics.

**Table S22.** **Directional pleiotropy and heterogeneity test of the instrumental SNPs for 10 psychiatric traits on mouth ulcers.**

| **Exposure** | **Outcome** | **MR-PRESSO global Test** | ***P*** | **Cochran’s *Q*** | **df** | ***P*** |
| --- | --- | --- | --- | --- | --- | --- |
| anxiety disorders | mouth ulcers | 1.20 | 0.875 | 0.67 | 3 | 0.881 |
| ADHD |  | 10.57 | 0.480 | 8.56 | 9 | 0.479 |
| ASD^$^ |  | 23.79 | 0.026 | 18.24 | 9 | 0.033 |
| ASD^&^ |  | 12.07 | 0.332 | 9.71 | 8 | 0.286 |
| BIP |  | 29.52 | 0.093 | 26.61 | 18 | 0.087 |
| insomnia |  | 233.41 | 0.075 | 231.15 | 201 | 0.071 |
| MDD |  | 9.88 | 1.000 | 9.25 | 27 | 0.999 |
| mood instability |  | 39.17 | 0.072 | 36.18 | 25 | 0.069 |
| neuroticism |  | 49.93 | 0.016 | 46.44 | 28 | 0.016 |
| SCZ |  | 173.93 | <0.0001 | 170.72 | 104 | 4.08×10^-5^ |
| subject wellbeing |  | 32.49 | 0.600 | 30.60 | 33 | 0.587 |

$, using 10 instrumental SNPs to perform MR-PRESSO global test and Cochran’s *Q* test; &, excluding the pleiotropic SNP (rs910805) and using remained 9 instrumental SNPs to perform MR-PRESSO global test and Cochran’s *Q* test; ADHD, attention deficit/hyperactivity disorder; ASD, autism spectrum disorder; BIP, bipolar disorder; MDD, major depressive disorder; SCZ, schizophrenia; MR-PRESSO, mendelian randomization pleiotropy residual sum and outlier.

**Table S23. Directional pleiotropy and heterogeneity test of the instrumental variables for mouth ulcers on 8 psychiatric traits.**

| **Exposure** | **Outcome** | **MR-PRESSO global Test** | ***P*** | **Cochran’s *Q*** | **df** | ***P*** |
| --- | --- | --- | --- | --- | --- | --- |
| mouth ulcers | ADHD | 78.28 | 0.142 | 76.07 | 64 | 0.143 |
|  | ASD | 51.16 | 0.893 | 49.63 | 63 | 0.890 |
|  | BIP | 65.83 | 0.281 | 63.84 | 58 | 0.279 |
|  | insomnia | 82.05 | 0.041 | 78.91 | 59 | 0.043 |
|  | MDD | 40.03 | 0.933 | 38.56 | 53 | 0.932 |
|  | neuroticism | 53.27 | 0.537 | 51.28 | 53 | 0.541 |
|  | SCZ | 76.57 | 0.075 | 74.23 | 58 | 0.074 |
|  | subject wellbeing | 34.00 | 0.249 | 31.12 | 27 | 0.266 |

ADHD, attention deficit/hyperactivity disorder; ASD, autism spectrum disorder; BIP, bipolar disorder; MDD, major depressive disorder; SCZ, schizophrenia; MR-PRESSO, mendelian randomization pleiotropy residual sum and outlier.
